# Supplementary material for: Core and accessory genomic traits of Vibrio cholerae O1 drive lineage transmission and disease severity
Source: Nat Commun. 2024 Sep 23;15:8231. doi: 10.1038/s41467-024-52238-0 (PMC11420230; doi:10.1038/s41467-024-52238-0)
Supplement: Supplementary file 1 — Supplementary Information [file 41467_2024_52238_MOESM1_ESM.pdf]

Supplementary Materials for

Core and accessory genomic traits of *Vibrio Cholerae* O1 drive lineage transmission and disease severity

Alexandre Maciel-Guerra *et al*

*Corresponding author. Email: tania.dottorini@nottingham.ac.uk

This PDF file includes:

- **Supplementary Figures:** Figs. S1 to S23
- **Supplementary Tables:** Table S1
- **Supplementary Notes:** 1 to 6

Supplementary Figures

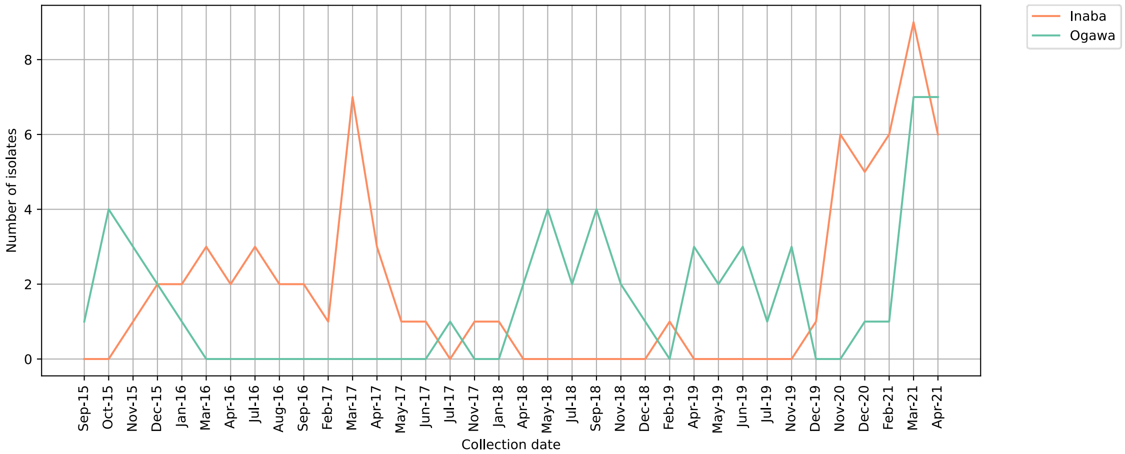

Figure S1. Temporal distribution of *Vibrio cholerae* serotypes from 2015 to 2021. The graph shows the serotype (Ogawa and Inaba) distribution of *V. cholerae* isolates from Bangladesh for 129 isolates. The Ogawa and Inaba serotypes of *V. cholerae* are indicated in green and orange, respectively.

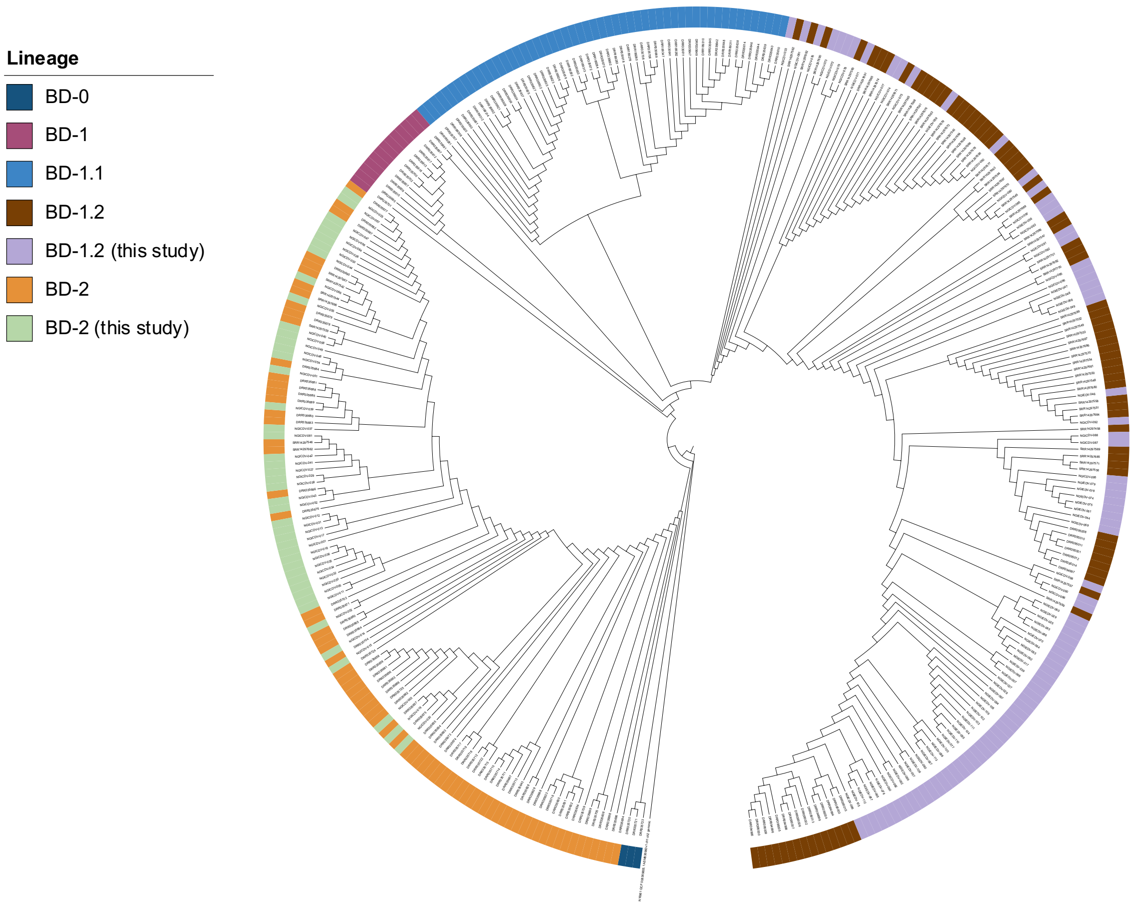

Figure S2. Maximum likelihood phylogenetic tree based on the core genome of the whole cohort of 129 *V. cholerae* El tor O1 (our study) together with 218 isolates collected in Bangladesh from 2004 to 2022 (The European Nucleotide Archive-ENA (http://www.ebi.ac.uk/ena). The reference genome *V. cholerae* N16961 was used as an outgroup. Branches with less than 95% ultrafast bootstrap were deleted. The outer coloured ring of the tree indicates the lineage of the Bangladesh sourced isolates.

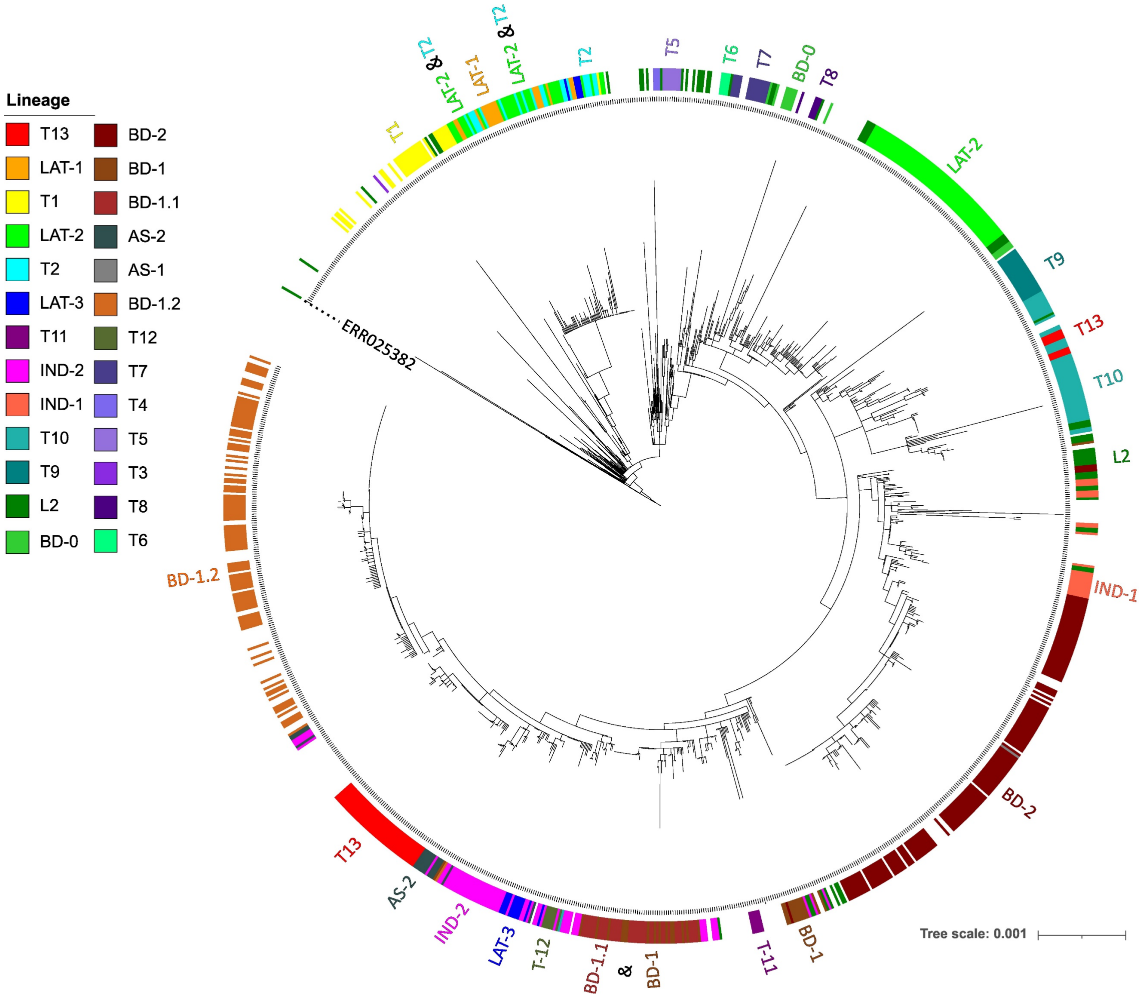

Figure S3. **SNP based maximum likelihood tree of *V. cholerae* O1 El Tor strains collected across Africa, Asia, America and Europe between 1957 and 2022.** 1134 *V. cholerae* O1 El Tor strains isolated across 84 different countries from Africa, Asia, America and Europe between 1957 and 2022 were considered in the analysis. Reference genome (VC N16961-NC_002505.1; NC_002506.1). The tree is rooted at the Outgroup (Indonesian sample -ERR025382). Branches with less than 95% ultrafast bootstrap support were deleted.

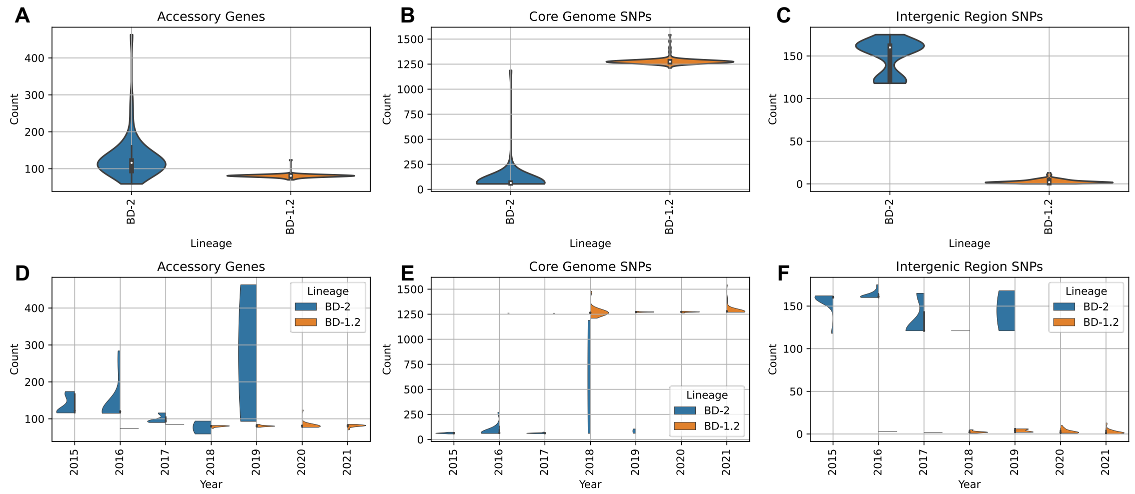

Figure S4. Violin plots showing the count of accessory genes, core genome SNPs and intergenic SNPs for (**A-C**) the two lineages (BD-1.2 and BD-2) and (**D-F**) over the different collection years (2015 to 2021). The violin plots show the distribution of the data, with each distribution representing one lineage or year. Inside each violin plot is a box plot, with the box showing the interquartile range (IQR), the whiskers showing the rest of the distribution as a proportion of 1.5 x IQR and the white circle representing the median value.

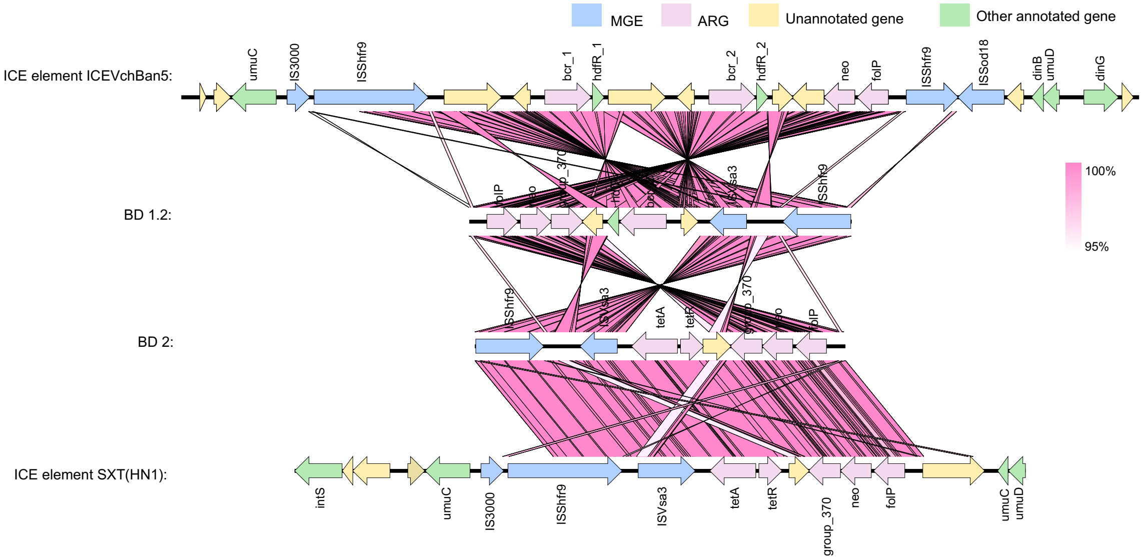

Figure S5. **BD1.2 and BD2 comparative contigs maps.** The positions of the MGEs (blue) ARGs (pink), unannotated genes (yellow) and other annotated genes, not ARGs, (green) are shown on an ideogram of the two ICE elements ICEVchBan5 and SXT (HN1) in BD1.2 and BD2, respectively. The 5'-3'orientation of each element is shown by an arrow. Genomic (amino acid) similarity is measured by tblastx and is shown as pink lines between sequences.

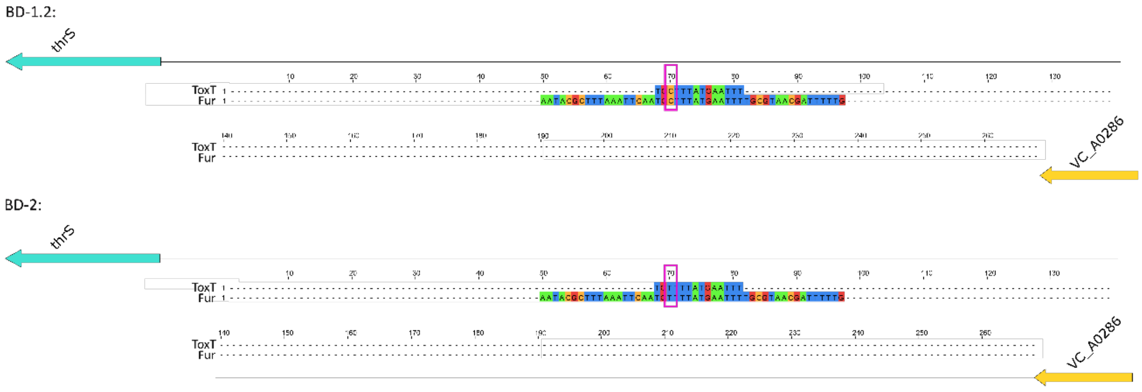

Figure S6. The non-synonymous intergenic SNP (C70T), located within the transcription factor binding sites (TFBSs) of *ToxT* and *Fur,* exhibits significant allelic distribution between BD-1.2 and BD-2 lineages. The SNP (C70T), indicated by a pink box, is located in the TFBSs of *ToxT* (89-102 bp upstream *thrS*) and *Fur* (50-97 bp upstream *thrS*) between the genes *thrS* and *VC_A0286*. The C variant, highlighted in orange, at position 70bp (third last nucleotide of *ToxT* TFBS and twenty-eighth nucleotide within the *Fur* TFBS) is uniquely present in the BD-1.2 lineage (top panel) whereas, all BD-2 isolates (bottom panel) exhibit a T at this position.

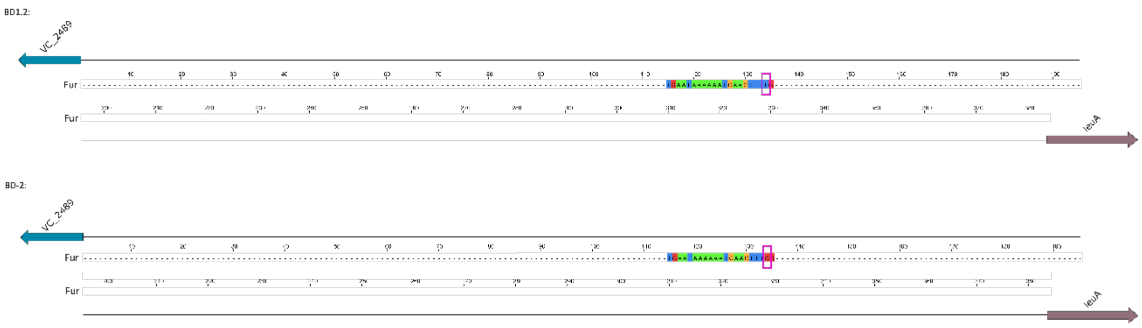

Figure S7. The non-synonymous intergenic SNP (T134G), located within the TFBS of *Fur*, exhibits significant allelic distribution between BD-1.2 and BD-2 lineages. The SNP (T134G), indicated by a pink box, is located in the TFBS of Fur (115-135bp upstream *VC_2489*) between the genes *VC_2489* and *VC_2490* (*leuA*). The T variant, highlighted in blue at position 134bp (second last nucleotide in *Fur* TFBS) is uniquely present in the BD-1.2 lineage (top panel) whereas, all BD-2 isolates (bottom panel) exhibit a G in this position.

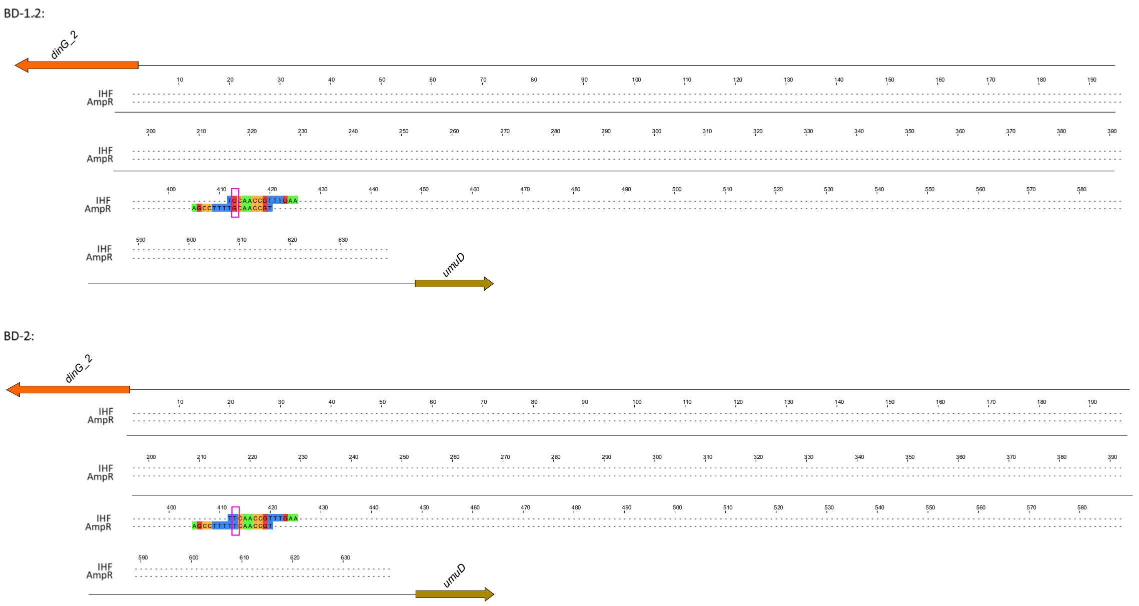

Figure S8. The non-synonymous intergenic SNP (G413T), located within the TFBSs of *IHF* and *AmpR*, exhibits significant allelic distribution between BD-1.2 and BD-2 lineages. The SNP (G413T), indicated by a pink box, is located in the TFBSs of *IHF* (412-425 bp upstream *dinG*) and *AmpR* (405-420bp upstream *dinG*) between the genes *dinG* and *umuD*. The G variant, highlighted in red, at position 413bp (second nucleotide of *IHF* TFBS and ninth nucleotide within the *AmpR* TFBS) is uniquely present in the BD-1.2 lineage (top panel) whereas, all BD-2 isolates (bottom panel) exhibit a T at this position.

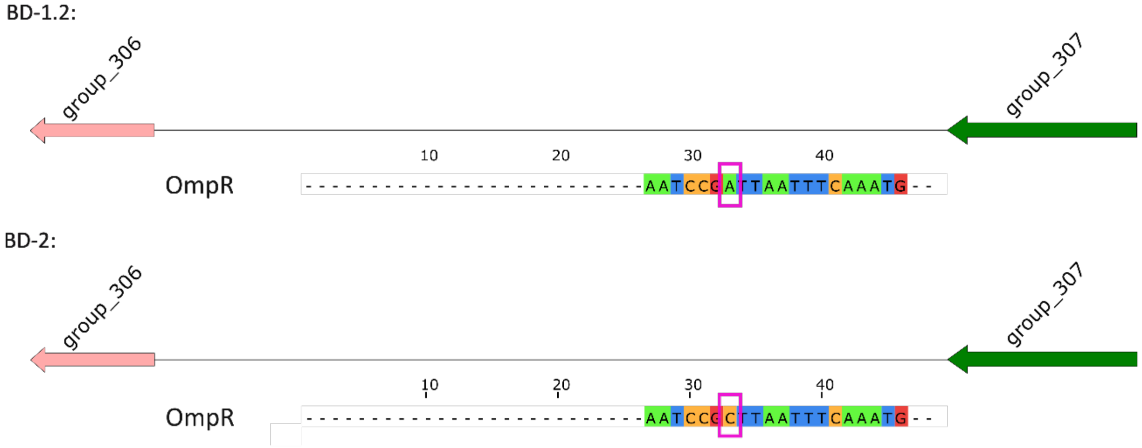

Figure S9. The non-synonymous intergenic SNP (A33C), located within the TFBS of *OmpR,* exhibits significant allelic distribution between BD-1.2 and BD-2 lineages. The SNP (A33C), indicated by a pink box, is located in the TFBS of *OmpR* (27-46 bp upstream *group_306*) between the genes *group_306* and *group_307*. The A variant, highlighted in green, at position 33bp (seventh nucleotide of *OmpR* TFBS) is uniquely present in the BD-1.2 lineage (top panel) whereas, all BD-2 isolates (bottom panel) exhibit a C at this position.

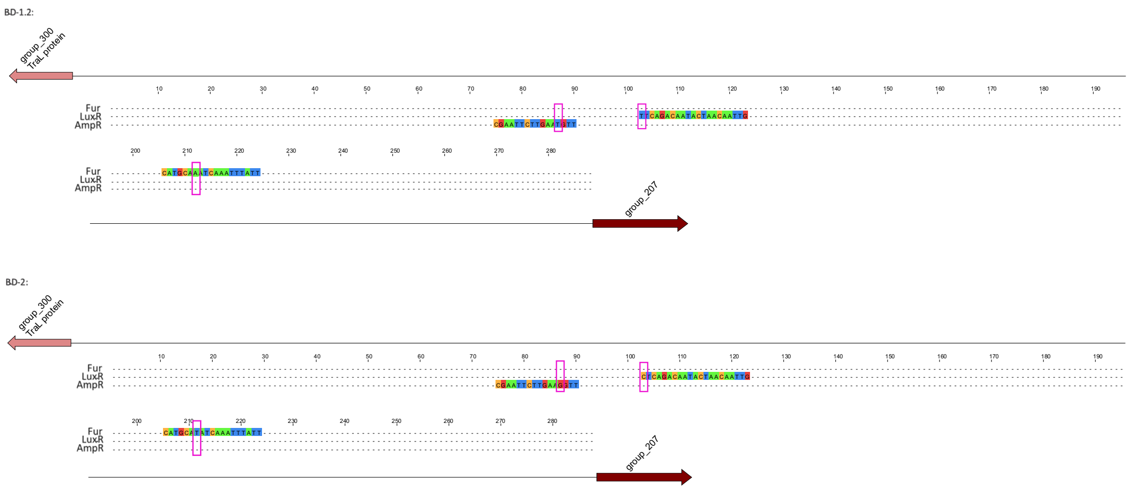

Figure S10. The non-synonymous intergenic SNPs (T87G, T103C, A212T), located within the TFBSs of *AmpR, LuxR* and *Fur* respectively, exhibit significant allelic distribution between BD-1.2 and BD-2 lineages. The SNP (T87G), indicated by a pink box, is located in the TFBSs of *AmpR* (75-90 bp upstream *group_300*) between the genes *group_300* and *group_207*. The T variant, highlighted in blue, at position 87bp (fourth last nucleotide of *AmpR*) is uniquely present in the BD-1.2 lineage (top panel) whereas, all BD-2 isolates (bottom panel) exhibit a G at this position. The SNP (T103C), indicated by a pink box, is located in the TFBSs of *LuxR* (103-123 bp upstream *group_300*) between the genes *group_300* and *group_207*. The T variant, highlighted in blue, at position 103bp (first nucleotide of *LuxR*) is uniquely present in the BD-1.2 lineage (top panel) whereas, all BD-2 isolates (bottom panel) exhibit a C at this position. The SNP (A212T), indicated by a pink box, is located in the TFBSs of *Fur* (206-224 bp upstream *group_300*) between the genes *group_300* and *group_207*. The A variant, highlighted in green, at position 212bp (seventh nucleotide of *Fur*) is uniquely present in the BD-1.2 lineage (top panel) whereas, all BD-2 isolates (bottom panel) exhibit a T at this position.

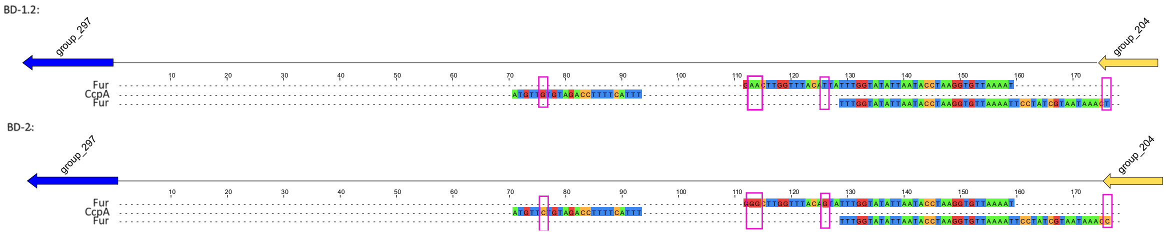

Figure S11. The non-synonymous intergenic SNPs (G76C, A113G, A114G, T126G, and T176C), located within the TFBSs of *CcpA* and *Fur,* exhibit significant allelic distribution between BD-1.2 and BD-2 lineages. The SNP (G76C), indicated by a pink box, is located in the TFBS of *CcpA* (71-93 bp upstream *group_297*) between the genes *group_297* and *group_204*. The G variant, highlighted in red, at position 76bp (fifth nucleotide of *CcpA* TFBS) is uniquely present in the BD-1.2 lineage (top panel) whereas, all BD-2 isolates (bottom panel) exhibit a T at this position. The SNPs (A113G, A114G, T126G, and T176C), indicated by pink boxes, are located in the TFBSs of *Fur* (112-159 bp and 129-176 bp upstream *group_297*) between the genes *group_297* and *group_204*. The A variants, highlighted in green, and T variants, highlighted in blue at positions 113bp ,114bp ,126bp and 176 bp (second, third, fifteenth and last nucleotides of *Fur* TFBS) are uniquely present in the BD-1.2 lineage (top panel) whereas, all BD-2 isolates (bottom panel) exhibit a G or C at this position.

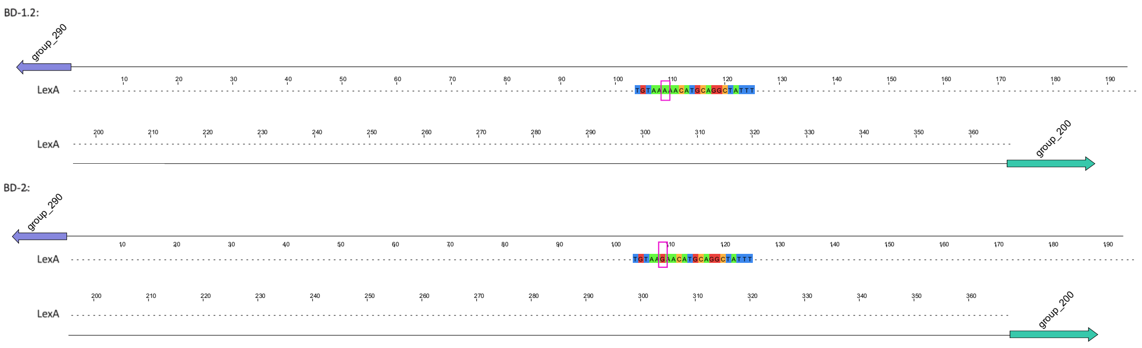

Figure S12. The non-synonymous intergenic SNP (A109G), located within the TFB of *LexA,* exhibits significant allelic distribution between BD-1.2 and BD-2 lineages. The SNP (A109G), indicated by a pink box, is located in the TFBS of *LexA* (104-125 bp upstream *group_290*) between the genes *group_290* and *group_200*. The A variant, highlighted in green, at position 109bp (fifth nucleotide of *LexA* TFBS) is uniquely present in the BD-1.2 lineage (top panel) whereas, all BD-2 isolates (bottom panel) exhibit a G at this position.

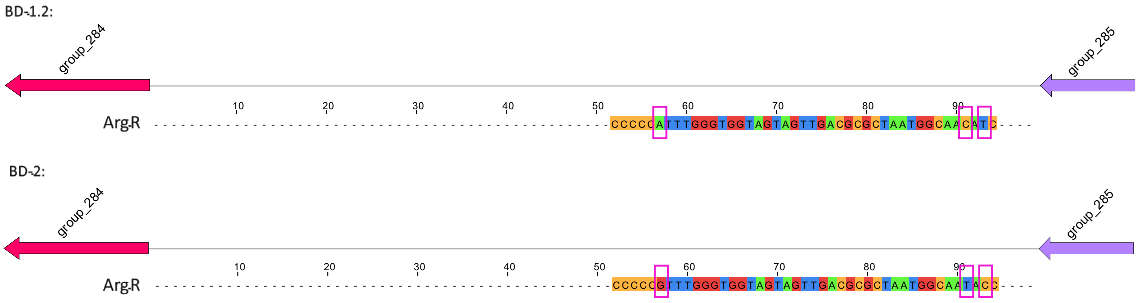

Figure S13. The non-synonymous intergenic SNPs (A57G, C91T, T93C), located TFBS of *ArgR,* exhibits significant allelic distribution between BD-1.2 and BD-2 lineages. The SNPs (A57G, C91T, T93C), indicated by pink boxes, are located in the TFBSs of *ArgR* (52-94 bp upstream *group_284*) between the genes *group_284* and *group_285*. The A variant, highlighted in green, at position 57bp (fifth last nucleotide of *ArgR* TFBS) is uniquely present in the BD-1.2 lineage (top panel) whereas, all BD-2 isolates (bottom panel) exhibit a G at this position. The C variant, highlighted in orange, at position 91bp (fourth last nucleotide of *ArgR* TFBS) is uniquely present in the BD-1.2 lineage (top panel) whereas, all BD-2 isolates (bottom panel) exhibit a T at this position. The T variant, highlighted in blue, at position 93bp (second last nucleotide of *ArgR* TFBS) is uniquely present in the BD-1.2 lineage (top panel) whereas, all BD-2 isolates (bottom panel) exhibit a C at this position.

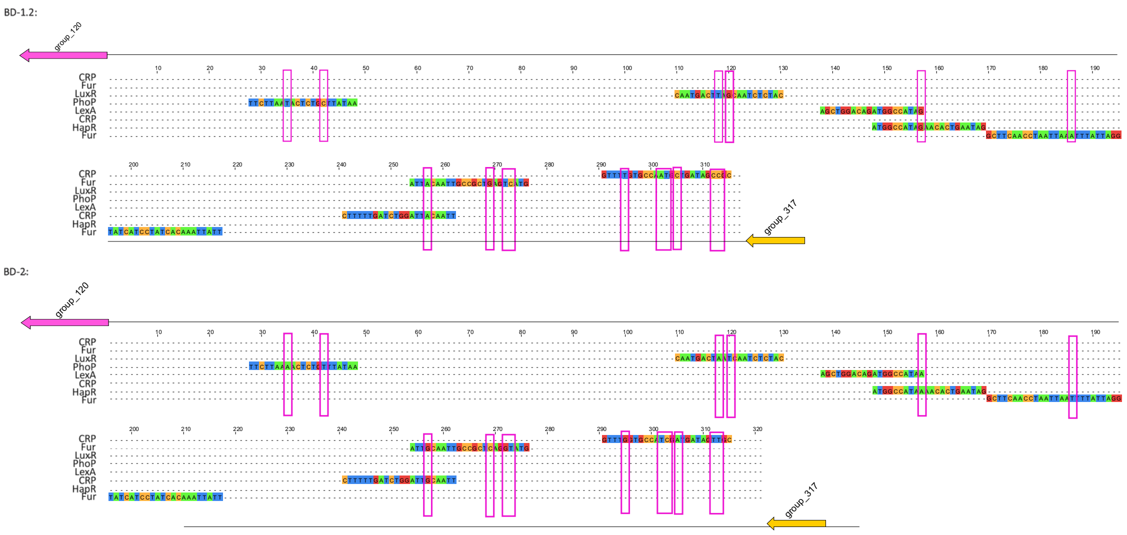

Figure S14. The non-synonymous intergenic SNP (T35A, C42T, T118A, G120T, G157A, A186T, A257G, G269C, T272G, C273T, T295G, A302T, T303C, C305A, C312T, C313T), located within the TFBSs of *CRP, Fur, LuxR, PhoP, LexA* and *HapR,* exhibit significant allelic distribution between BD-1.2 and BD-2 lineages. Two SNPs (T35A, C42T), indicated by pink boxes, are located in the TFBSs of *PhoP* (28-48 bp upstream *group_120*) between the genes *group_120* and *group_317*. The T35 and C42 variants, highlighted in blue and orange, at positions 35bp (eighth nucleotide of *LuxR*) and 42 bp (seventh last nucleotide of *LuxR*) are uniquely present in the BD-1.2 lineage (top panel) whereas, all BD-2 isolates (bottom panel) exhibit a A(35bp) or T(42bp) at these positions. Two SNPs (T118A, G120T), indicated by pink boxes, are located in the TFBS of *LuxR* (110-130 bp upstream *group_120*) between the genes *group_120* and *group_317*. The T118 and G120 variants, highlighted in blue and red, at positions 118bp (ninth nucleotide of *LuxR*) and 120 bp (eleventh last nucleotide of *LuxR*) are uniquely present in the BD-1.2 lineage (top panel) whereas, all BD-2 isolates (bottom panel) exhibit a A(118bp) or T(120bp) at these positions. The SNP (G157A), indicated by a pink box, is located in the TFBSs of *LexA* (138-157 bp upstream *group_120*) and *HapR* (148-169bp upstream *group_120*) between the genes *group_120* and *group_317*. The G variant, highlighted in red, at position 157bp (last nucleotide of *LexA* TFBS and tenth nucleotide within the *HapR* TFBS) is uniquely present in the BD-1.2 lineage (top panel) whereas, all BD-2 isolates (bottom panel) exhibit an A at this position. The SNP (A257G), indicated by a pink box, is located in the TFBSs of *Fur* (254-276 bp upstream *group_120*) and *Crp* (241-262bp upstream *group_120*) between the genes *group_120* and *group_317*. The A variant, highlighted in green, at position 257bp (fourth nucleotide of *Fur* TFBS and thirteenth nucleotide within the *Crp* TFBS) is uniquely present in the BD-1.2 lineage (top panel) whereas, all BD-2 isolates (bottom panel) exhibit a G at this position. Three SNPs (A186T, G269C, T272G), indicated by pink boxes, are located in the TFBS of *Fur* (170-217bp and 254-276bp upstream *group_120*) between the genes *group_120* and *group_317*. The A186, G269 and T272 variants, highlighted in green, red and blue, are uniquely present in the BD-1.2 lineage (top panel) whereas, all BD-2 isolates (bottom panel) exhibit a T(186bp) ,C(269bp) or G (272 bp) at these positions. Six SNPs (T295G, A302T, T303C, C305A, C312T, C313T), indicated by pink boxes, are located in the TFBS of *Crp* (291-315bp upstream *group_120*) between the genes *group_120* and *group_317*. The T295, A302 and T303, C305, C312 and C313 variants, are uniquely present in the BD-1.2 lineage (top panel) whereas, all BD-2 isolates (bottom panel) exhibit a T(302, 312 and 313 bp) , C(303bp) A (305bp) or G (295 bp) at these positions.

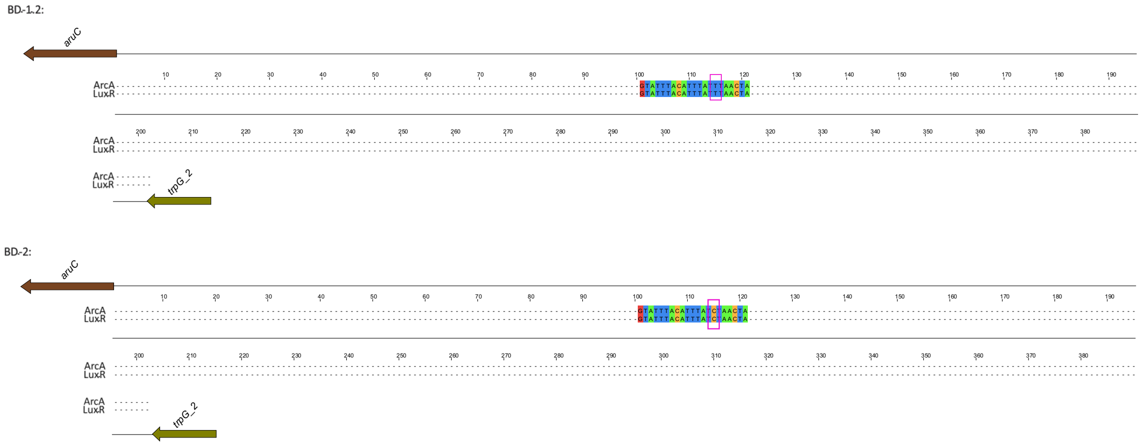

Figure S15. The non-synonymous intergenic SNP (T115C), located within the TFBSs of *ArcA* and *LuxR*, exhibits significant allelic distribution between BD-1.2 and BD-2 lineages. The SNP (T115C), indicated by a pink box, is located in the TFBSs of *ArcA* (101-121 bp upstream *aruC*) and *LuxR* (101-121 bp upstream *aruC*) between the genes *aruC* and *trpG*. The T variant, highlighted in blue, at position 115bp (seventh last nucleotide of *aruC* and *trpG_2* TFBSs) is uniquely present in the BD-1.2 lineage (top panel) whereas, all BD-2 isolates (bottom panel) exhibit a C at this position.

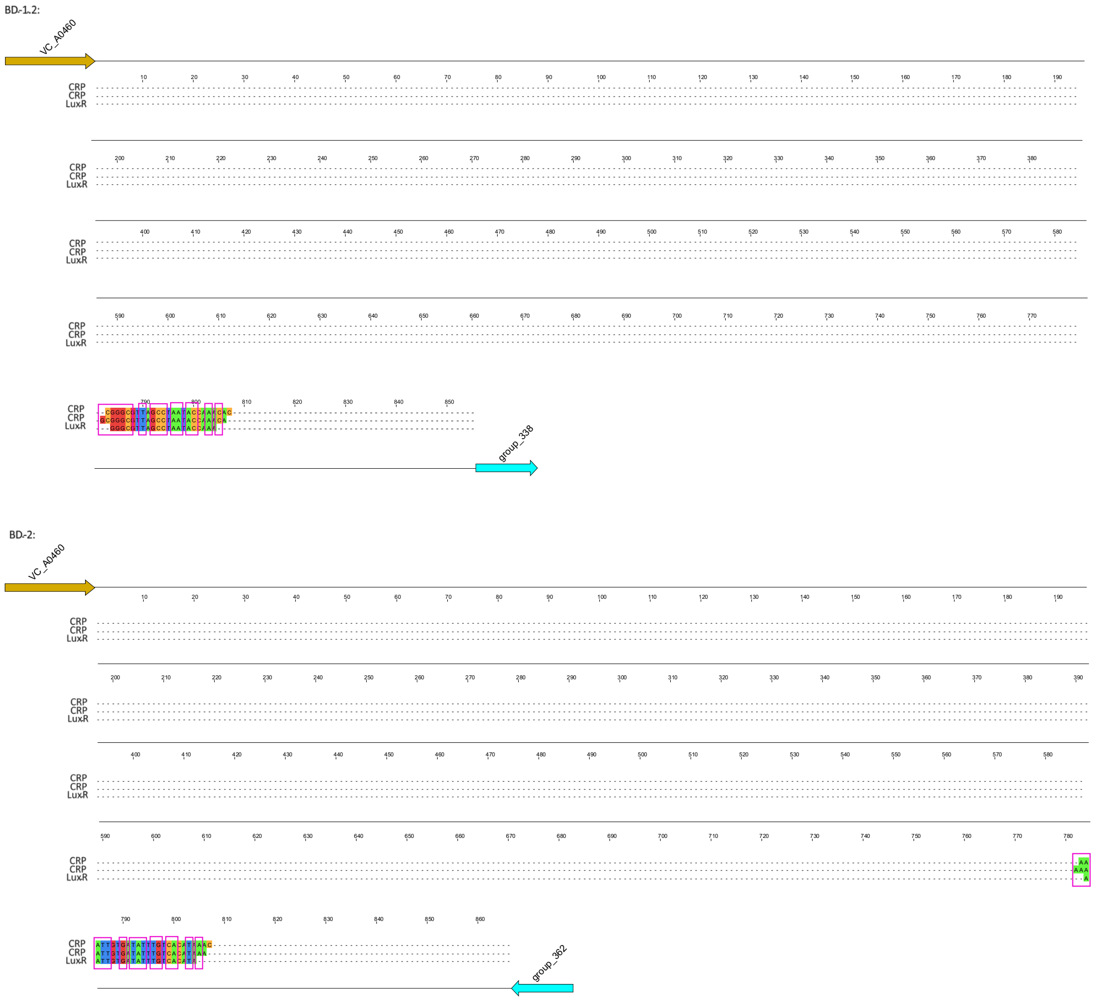

Figure S16. The non-synonymous intergenic SNPs (G782A, C783A, G784A, G785A, G786T, C787T, T790G, G792T, C793A, C794T, A796T, A797G, A799C, C800A, A803T, C805A), located within the TFBSs of *CRP* and *LuxR*, exhibits significant allelic distribution between BD-1.2 and BD-2 lineages. The SNPs (G782A, C783A, G784A, G785A, G786T, C787T, T790G, G792T, C793A, C794T, A796T, A797G, A799C, C800A, A803T, C805A), indicated by pink boxes, are located in the TFBSs of *CRP* (782-806 and 783-807 bp upstream *VC_A0460*) and *LuxR* (784-804bp upstream *VC_A0460*) between the genes *VC_A0460* and *group_338*. The variants G782, C783, G784, G785, G786, C787, T790, G792, C793, C794, A796, A797, A799, C800, A803T, C805, are uniquely present in the BD-1.2 lineage (top panel) whereas, 69% of BD-2 isolates (bottom panel) exhibit A782, A783, A784, A785, A786, T787, G790, T792, A793, T794, T796, G797, C799, A800, T803, A805 in these positions.

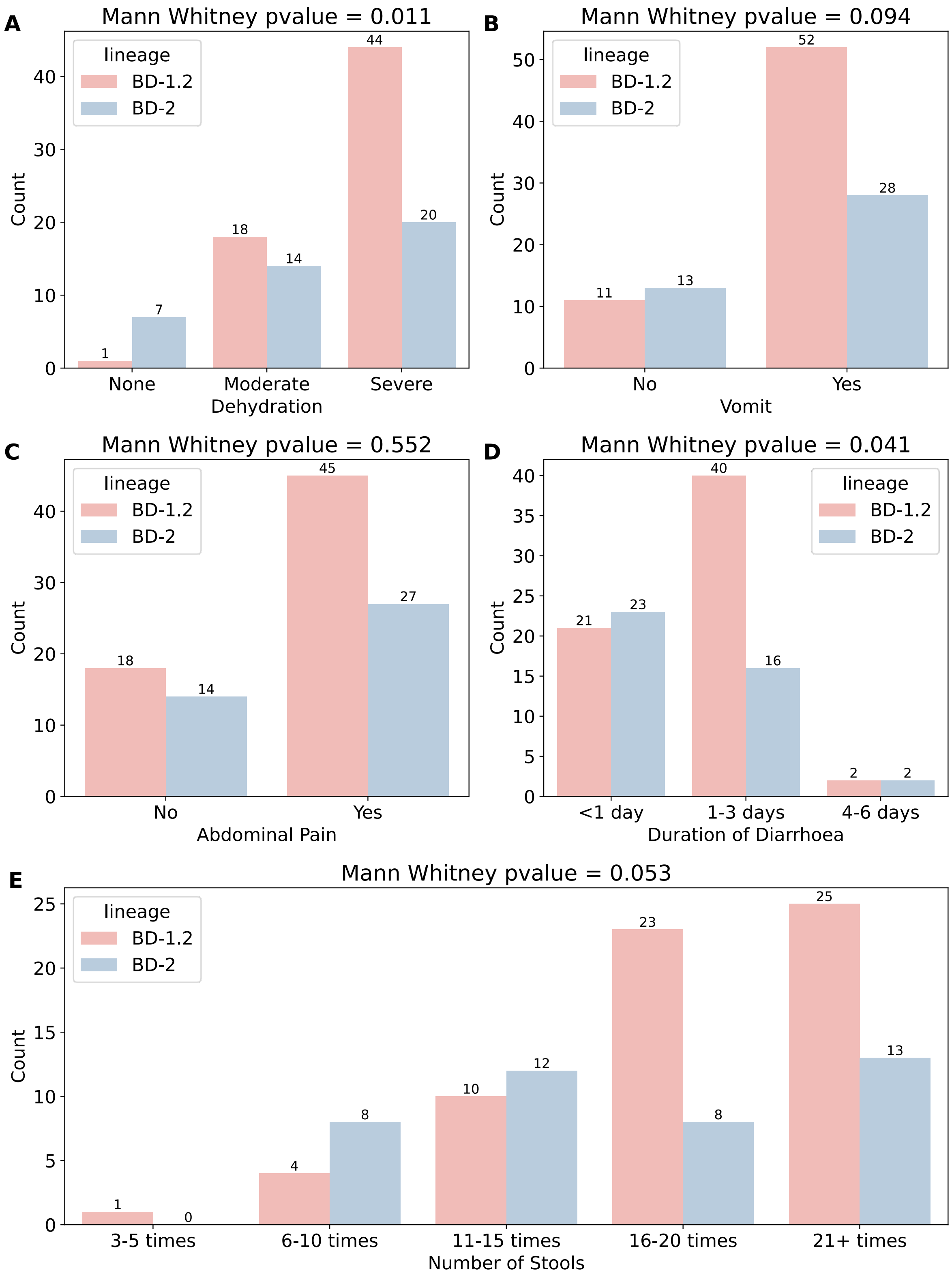

Figure S17. Bar plots indicating the overall count of each category in each symptom over the lineages BD-1.2 and BD-2. (A) Dehydration, (B) Vomit, (C) Abdominal Pain, (D) Duration of Diarrhoea, (E) Number of Stools. Two-tailed Mann Whitney U tests were used to assess for statistical differences between lineages for each symptom.

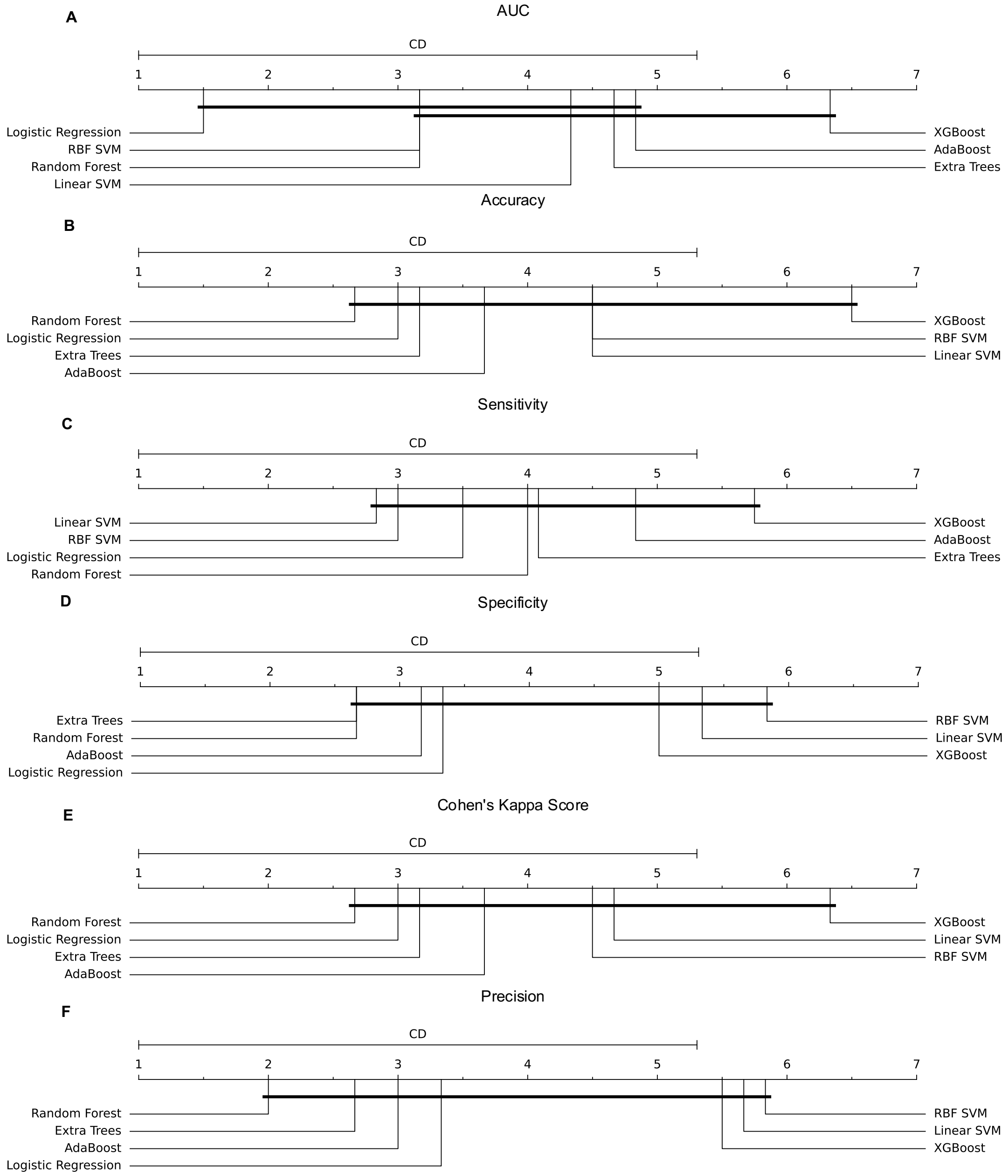

Figure S18. Nemenyi *post-hoc* tests. Comparison of the performance of the 5 classifiers and 2 meta-methods, using their average ordinal rank over the clinical symptom analysed based on six performance metrics (A) AUC, (B) accuracy, (C) sensitivity, (D) specificity, (E) Cohen's kappa score and (F) precision for *V. cholerae*. The x-axis indicates the average ordinal rank of the machine learning methods. The scale is from 1 (best rank) to 7 (worst rank). The ordinal rank of a classifier is defined as follows: the ML method with the best AUC is given rank 1, the second-best AUC rank 2 and the *n*-th AUC best rank *n*, with *n* being the number of machine learning methods used. For each clinical symptom, the methods are ranked between 1 (highest AUC) and 7 (lowest AUC), since in this case there are 7 machine learning methods used. Next, for each method, the ranks are averaged based on the six clinical symptoms studied. The critical distance (CD) is defined based on the Nemenyi *post-hoc* test, all the methods that fall in the same bold bar below the axis are considered statistically equivalent based on the CD value.

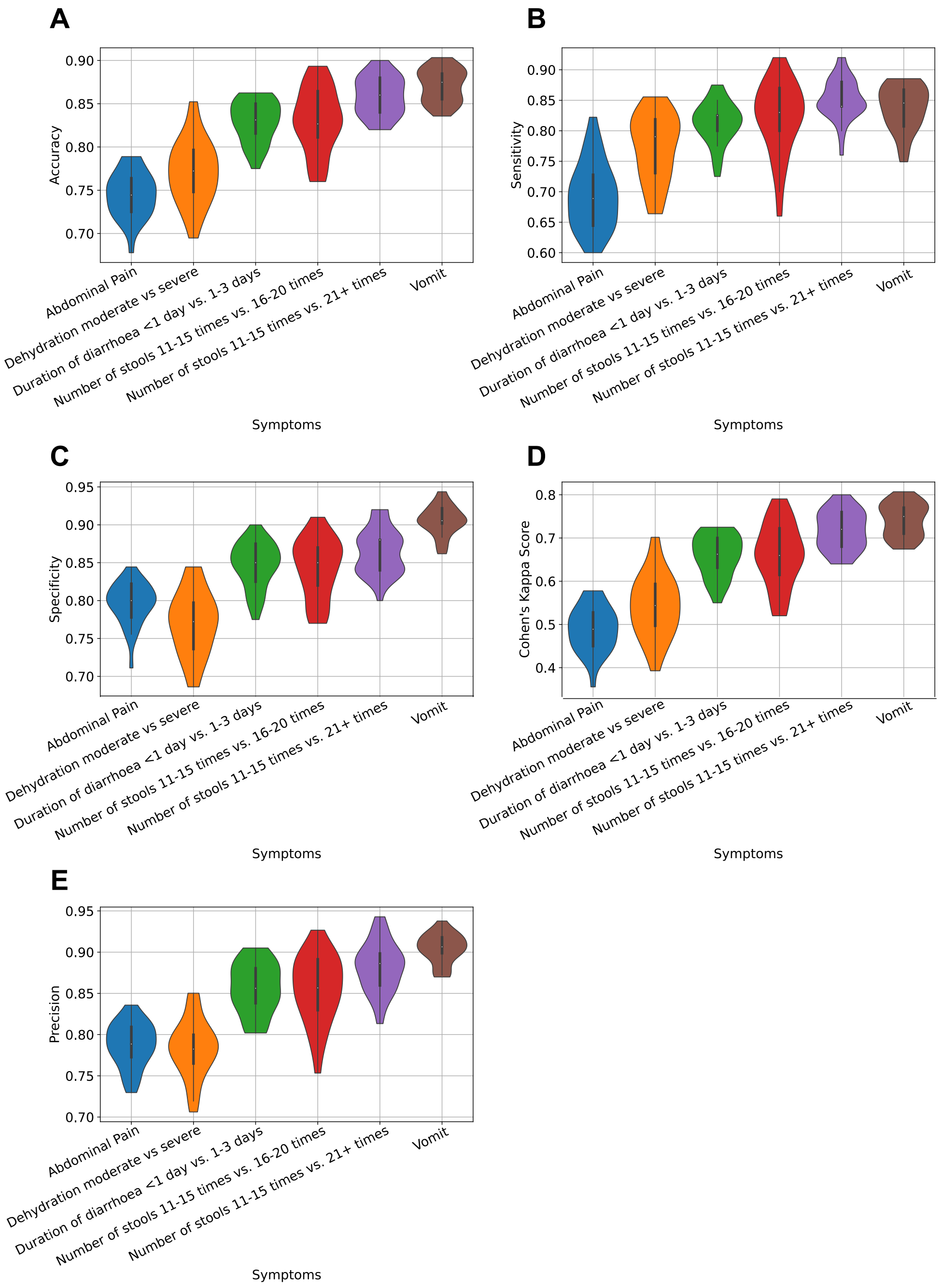

Figure S19. A supervised machine learning pipeline successfully predicts the clinical manifestations of hospitalized patients based on genomic determinants extracted from BD-1.2 isolates obtained from those same patients during hospitalization. Machine learning performance results for four performance indicators: (A) accuracy, (B) sensitivity, (C) specificity, (D) Cohen's Kappa Score and (E) precision from 30 training runs for each clinical symptom. The results shown are for the best classifier Logistic Regression, as defined by the Nemenyi test (**Fig. S18**). Predictive models were generated for six different clinical symptoms (X axis): abdominal pain; dehydration "moderate" vs "severe"; duration of diarrhoea <1 day vs. 1-3 days; number of stools 11-15 times vs. 16-20 times; number of stools 11-15 times vs. 21+ times; and vomit. The violin plots show the distribution of the data, with each distribution representing classification model. Inside each violin plot is a box plot, with the box showing the interquartile range (IQR), the whiskers showing the rest of the distribution as a proportion of 1.5 x IQR and the white circle representing the median value.

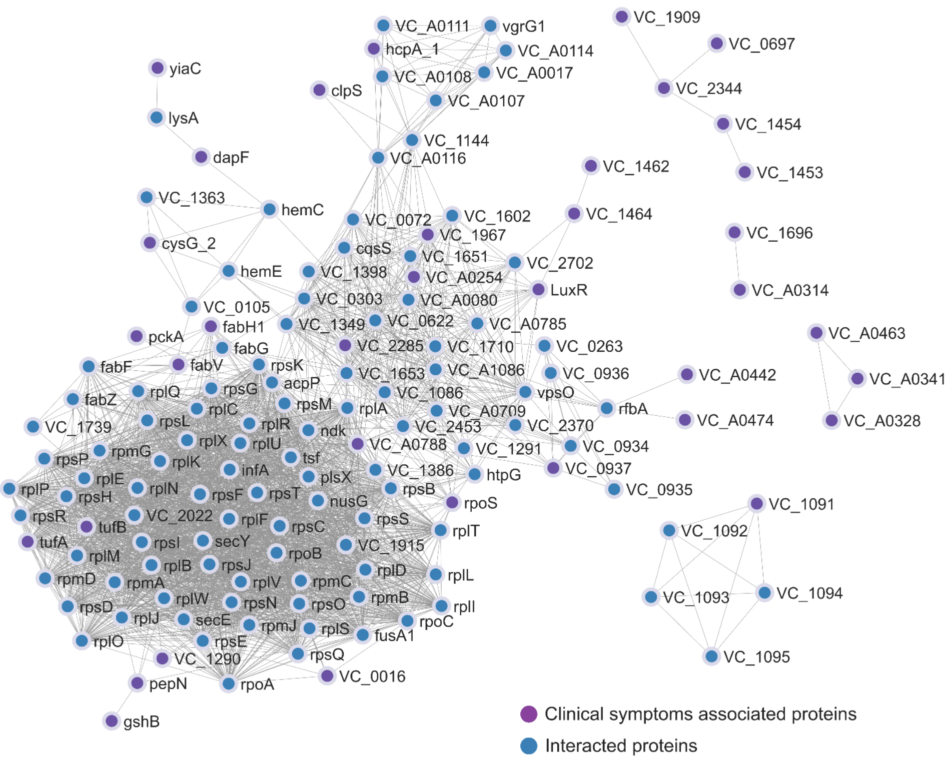

Figure S20. Protein-protein interaction (PPI) network of the interactome found to be associated with clinical symptoms The PPI network shows the proteins encoded by the genes predicted by the ML classifiers (36 proteins, nodes labelled in purple) to be strongly associated to the different clinical symptoms with 109 other interacting proteins (blue labelled nodes).

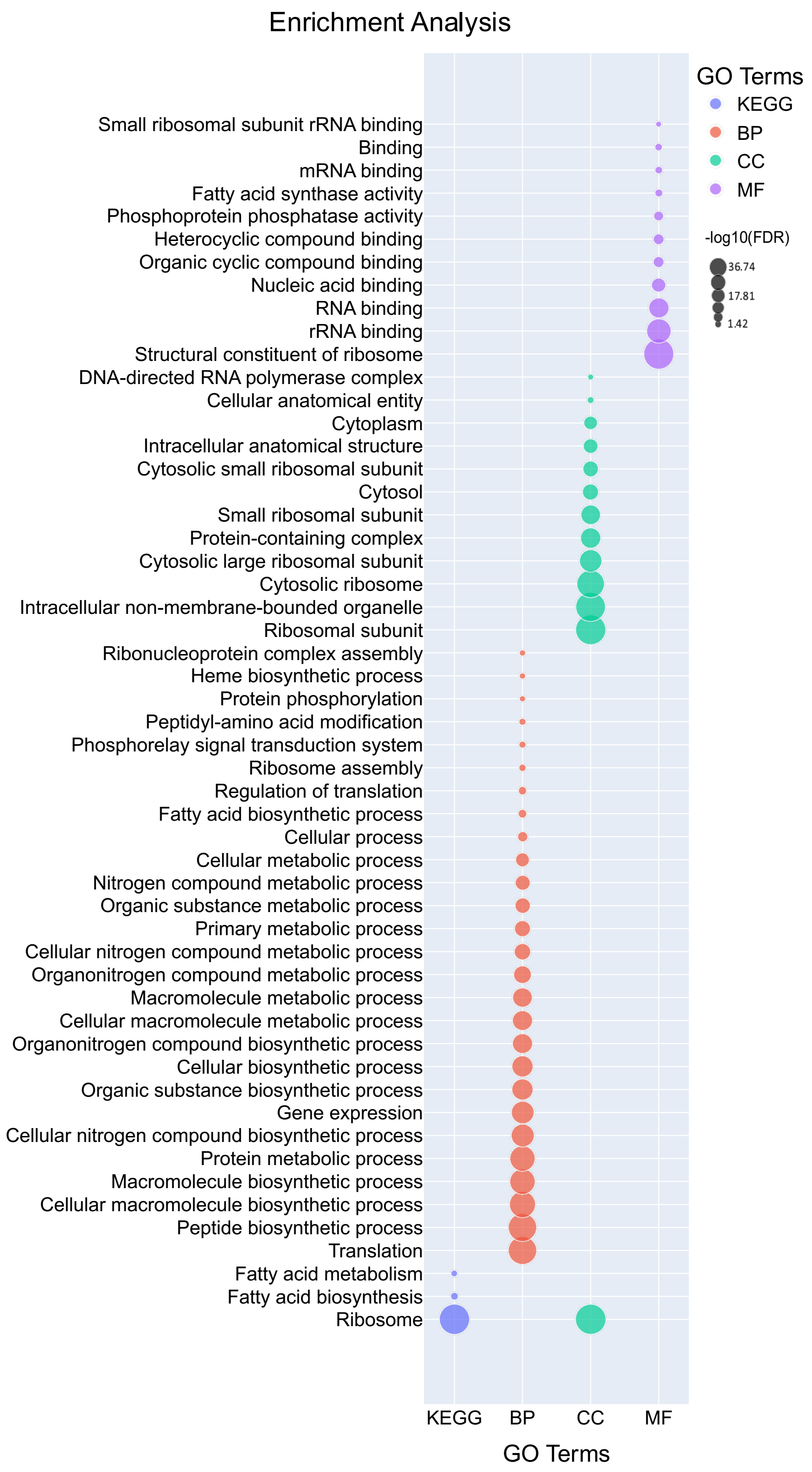

Figure S21. Gene set enrichment analysis of the 36 clinical symptoms-associated genes and the 109 other interacting proteins (found by the PPI). Gene ontology enrichment (molecular function (MF) in purple, cellular component (CC) in green and biological process (BP) in orange) and KEGG pathways analysis results (purple) are shown.

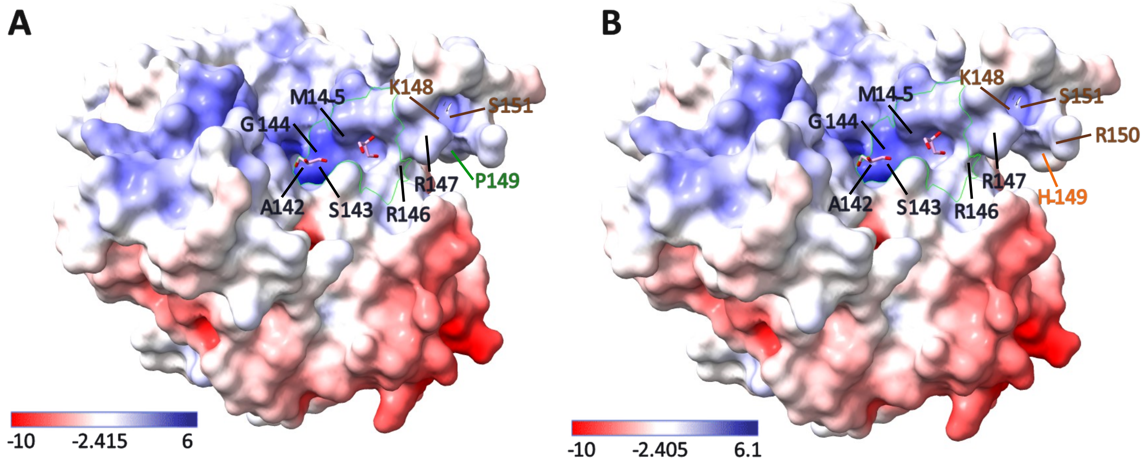

Figure S22. Representation of electrostatic surface potential of FabV allelic variants underlying BD-1.2 and BD-2 lineage evolution and clinical symptoms. A) Pro149 is highlighted in green. Amino acid residues (Lys148, Ser151, and Trp159) interacting with Pro149 (green) are shown in sticks models and coloured in brown, amino acids within 5Å vicinity are labelled in black amino. **B)** His149 is highlighted in orange, amino acid residues (Lys148, Ser151, and Trp159) interacting with Pro149 are shown in sticks models and coloured in brown, amino acids within 5Å vicinity are labelled in black amino. Molecular graphics and analyses performed with UCSF ChimeraX, developed by the Resource for Biocomputing, Visualization, and Informatics at the University of California, San Francisco, with support from National Institutes of Health R01-GM129325 and the Office of Cyber Infrastructure and Computational Biology, National Institute of Allergy and Infectious Diseases[1].

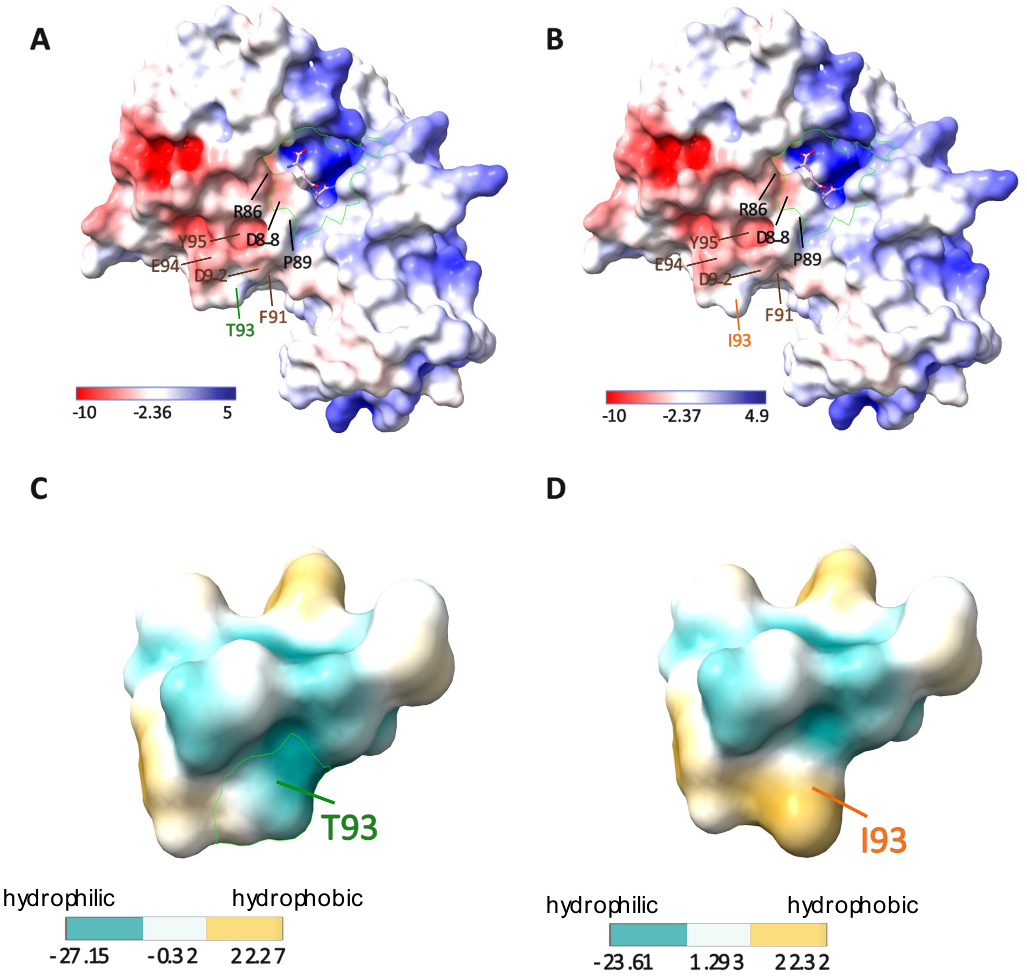

Figure S23. Representation of electrostatic surface potential of GshB allelic variants underlying BD-1.2 and BD-2 lineage evolution and clinical symptoms. A) Thr93 is highlighted in green, while amino acids interacting with Pro149 are labelled in brown. The gsh ligand (D88) and GshB ligand binding aminoacids (R86 and P89) are labelled in black; **B)** Ile93 is highlighted in orange, while amino acids interacting with Ile149 are labelled in brown; **C)** GshB protein side-chain surface coloured by hydrophobicity in the presence of Thr93 amino-acid; **D)** GshB protein side-chain surface coloured by hydrophobicity in the presence of Ile93. Molecular graphics and analyses performed with UCSF ChimeraX, developed by the Resource for Biocomputing, Visualization, and Informatics at the University of California, San Francisco, with support from National Institutes of Health R01-GM129325 and the Office of Cyber Infrastructure and Computational Biology, National Institute of Allergy and Infectious Diseases[1].

Supplementary Tables

Table S1. Core genes with a significant different allelic distribution between BD-1.2 and BD-2. Core genes containing non-synonymous SNPs and showcasing the allelic variants that were found exclusively in one lineage but absent in the other lineage. For each SNP, the allelic frequency of the major allele and minor allele and the *p*-value (based on a two-sided Fisher exact test with Bonferroni correction), for the allelic distribution between BD-1.2 and BD-2 have been calculated. The reference allelic variation refers to the nucleotide present in reference genome sequence of *V. cholerae* N16961 El Tor (NCBI Accession ID: NC_002505.1 and NC_002506.1) and the alteration allelic variation refers to the nucleotide absent in the reference genome, as previously done by Monir *et al*[2].

Gene name	*Vibrio cholerae* name[a]	SNP genomic location	Alleles REF[b] / ALT[c]	Frequency of REF[b] allele in BD-1.2 / BD-2	Frequency of ALT[c] allele in BD-1.2 / BD-2	Amino-acid change	*p*-value (Fisher exact test)
appC	VC_1571[d]	676	G/A	0/100	100/0	Ala226Thr	7.97E-36
argG	VC_2642[d]	847	A/G	100/0	0/100	Thr283Ala	7.97E-36
bluF	VC_1641	448	C/T	0/100	100/0	Lys149Asn	7.97E-36
bluF	VC_1641	449	A/G	0/100	100/0	Ser150Arg	7.97E-36
bluF	VC_1641	451	T/G	0/100	100/0	Leu151his	7.97E-36
bluF	VC_1641	455	T/C	0/100	100/0	Gly152Ser	7.97E-36
bluF	VC_1641	456	G/A	0/100	100/0	Phe153Ala	7.97E-36
bluF	VC_1641	461	C/T	0/100	100/0	Gln154Ala	7.97E-36
bluF	VC_1641	462	A/G	0/100	100/0	Thr155Asn	7.97E-36
bluF	VC_1641	466	C/T	0/100	100/0	Ala156Ser	7.97E-36
bluF	VC_1641	469	C/T	0/100	100/0	Ile157Arg	7.97E-36
bluF	VC_1641	472	G/A	0/100	100/0	Asp158Ile	7.97E-36
clcA	VC_A0526[d]	311	G/A	0/100	100/0	Gly104Glu	7.97E-36
cobB	VC_1509[d]	149	C/T	100/0	0/100	Pro50Leu	7.97E-36
ctxB	VC_A0009	58	C/A	0/100	100/0	His20Asn	7.97E-36
cysG_1	VC_1363[d]	113	T/C	100/0	0/100	Val38Ala	7.97E-36
dltA_1	VC_A0149	3145	T/C	0/100	100/0	Phe1049Leu	7.97E-36

dsbD	VC_2701[d]	1748	C/T	0/100	100/0	Thr583Ile	7.97E-36
ftsI	VC_2407[d]	1472	G/A	0/100	100/0	Arg491His	7.97E-36
glmM	VC_0639[d]	587	G/T	100/0	0/100	Arg196Leu	7.97E-36
gyrA	VC_1258	1980	G/T	100/0	0/100	Asp660Glu	7.97E-36
hrpB	VC_0601	2345	C/T	100/0	0/100	Ala782Val	7.97E-36
licH	VC_1284[d]	166	G/A	0/100	100/0	Ala56Thr	7.97E-36
mak	VC0270[d]	346	G/A	0/100	100/0	Gly116Arg	7.97E-36
murI	VC_0158[d]	409	G/T	100/0	0/100	Ala137Ser	7.97E-36
mutL	VC_0345	1048	T/C	0/100	100/0	Cys350Arg	7.97E-36
nudF_2	VC_2435	325	C/T	100/0	0/100	Arg109Cys	7.97E-36
pctB_4	VC_0514	746	T/G	100/0	0/100	Leu249Trp	7.97E-36
phhA	VC_A0828[d]	56	A/T	100/0	0/100	Gln19Leu	7.97E-36
putA		1799	C/T	100/0	0/100	Ala600Val	7.97E-36
recD	VC_2319	2039	A/G	0/100	100/0	Tyr680Cys	7.97E-36
rssB_3	VC_1652	235	C/T	0/100	100/0	Leu79Phe	7.97E-36
skp	VC_2251	146	T/G	0/100	100/0	Leu46Trp	7.97E-36
suhB	VC_0745[d]	650	A/G	100/0	0/100	Glu217Gly	7.97E-36
tamA	VC_2548	797	C/T	100/0	0/100	Thr266Ile	7.97E-36
trmL_1	VC_A0627	16	A/T	100/0	0/100	Thr6Ser	7.97E-36
tyrS_1	VC_0631	1177	A/G	100/0	0/100	Thr393Ala	7.97E-36
valS	VC_2503	1796	G/A	0/100	100/0	Arg599His	7.97E-36
ycbB	VC_1268	976	C/T	100/0	0/100	Pro327Ser	7.97E-36

[a] *V. cholerae* name: specific VC (*Vibrio cholerae*) gene names.
[b] REF (Reference) allele: refers to the nucleotide present in reference genome sequence of *V. cholerae* N16961 El Tor (NCBI Accession ID: NC_002505.1 and NC_002506.1)
[c] ALT (Alteration) allele: refers to the nucleotide absent in the reference genome
[d] Metabolic genes found in the *V. cholerae* GSM model iAM-Vc960

Supplementary Notes

Supplementary Note 1:
The distribution of the counts of accessory genes in BD-1.2 and BD-2 was statistically different (Mann Whitney U test p value < 0.005, two-sided), Fig. S4. The mean number (±SD) of accessory genes in BD-1.2 was 81±6 compared to 130±63 in BD-2. Moreover, comparing any individual year from 2015 to 2016 (BD-2 lineage) with any individual year 2020 to 2021 (BD-1.2 lineage) exhibited a statistically significant difference in the distribution mean across all three genomic feature types, as determined by a Mann Whitney U test (p-value < 0.005, two-sided), Fig S4D-F, as expected due to the relationship between the lineage and the year of collection.

Supplementary Note 2:
The accessory genes *hlyA*, *mcrC*, and *mepM* were found in significantly more BD-2 isolates compared to BD-1.2. Hemolysin (*hlyA*) is a major virulence factor found in *V. cholerae* strains and it is known to initiate host apoptosis, contributing to diarrhoea[3]. The *mcrC* gene has been found within integrative and conjugative elements (ICE) in *V. cholerae,* which are associated with various bacterial adaptations such as antimicrobial resistance[4]. In addition, the *mepM_3* gene, exclusively present in all BD-2 isolates, encodes the murin DD-endopeptidase MepM protein, which is known to belong to the pathogenicity island VSP-2[5]. Conversely, the genes *lon* and *endA* were present in significantly more BD-1.2 isolates. Lon protease (*lon*) plays a significant role in regulating the motility and virulence several bacteria, including *V. cholerae*[6]. Extracellular endonuclease (*endA*) responsible for the control of biofilm extracellular DNA levels and the formation of the biofilm structure in *V. cholerae*[7].

Four genes (*mrr*, *tetA*, *tetR*, and *mcrC*) were exclusively found in the BD-2 lineage in both our cohort and the public BD sequences, see Supplementary Data 10**.** The genes *lon_3*, *bcr_2* and *hdfR_4* were absent in both our cohort and the public ENA isolates across all BD-2 samples in both our cohort and the ENA public sequences. The gene *hlyA_2* was present in 100% of our BD-2 isolates and absent in all of our BD-1.2 isolates, while in the ENA public sequences this gene was absent in all lineages, apart from BD-2 which had a presence of 97.78% (present in 44 of 45 isolates). The gene *aer_3* was present in 98.61% of our BD-2 isolates (present in 71 out of 72 isolates) and absent in all of our BD-1.2 isolates, while in the ENA public sequences this gene was absent in the lineages BD-1.1 and BD-1.2, and present in the lineages BD-0 (present in 3 out of 3 isolates), BD-1 (present in 1 out of 13 isolates) and BD-2 (present in 45 out of 45 isolates). Finally, the gene *endA* was absent in the BD-2 lineage within our cohort as well as the ENA public sequences while the gene *adh* was absent in our BD-2 isolates but present in only 4.17% of the ENA public BD-2 sequences.

Supplementary Note 3:
SNPs present in the genes *skp*, *tamA*, *clcA*, *valS*, *cysS*, *pepA* and *cysG* exhibited significantly different allelic distribution between BD-1.2 and BD-2 lineages in our cohort. Skp is a periplasmic chaperone involved in the type II secretion system, which is a pathway used by *V. cholerae* for the uptake of nutrients and to deliver toxins, such as cholera toxin, to target cells[8, 9]. The gene *tamA,* together with *tamB,* encodes components of a translocation and assembly module (Tam) which mediates the assembly of autotransporters, with Tam proteins contributing to the pathogenesis of several gram-

negative bacteria[10, 11]. The gene *clcA*, encodes an H^+/Cl^- transporter, that is reported to be linked to the acid tolerance response against hydrochloric acid[12]. *ClcA* is downregulated in alkaline environments but upregulated in low pH environments (e.g., the stomach) to facilitate its survival[12, 13]. The gene *valS* lies immediately downstream of the *pepA* and *holC* genes and it has been suggested by others that these three genes could be expressed as a polycistronic message from the *pepA* promoter[14, 15, 16]. The *pepA* gene has been identified as a negative transcriptional regulator of virulence determinants[16]; disruption of the *pepA* gene causes elevated expression of CT, *tcpA*, *toxT*, and *tcpP* specifically in response to variations in pH and temperature[14, 16]. The gene *cysG,* encoding precorrin-2 dehydrogenase, influences heme production, which affects virulence[17, 18, 19].

Supplementary Note 4

Using a combined pangenome may introduce biases due to the population structure of the underlying sequences. To address this concern, we conducted additional analyses:

Firstly, we constructed lineage-specific pangenomes for BD-1.2 and BD-2, comparing both core and accessory genes across each lineage.

Secondly, we repeated the analysis using isolates from our cohort along with publicly available BD-1.2 and BD-2 isolates. These analyses broadly confirmed our original results and are summarized below. However, it is important to note that while employing independent pangenomes addresses potential biases related to population structure, it also introduces several challenges within the framework of our comparative analysis. Specifically:

- (i) Gene naming consistency: The complex procedure for clustering genes relies on sequence similarity estimated across all gene sequences and gene synteny. When performing similarity and clustering analyses on separate datasets (i.e. separate lineages), as explained above with the Uniprot example *of hlyA* and *hlyA_1*, which share 99% protein sequence identity and are variants of the same gene, are treated as distinct gene entries despite being essentially the same gene. For example, consider a conserved gene, geneX, with two highly similar variants, geneX_A and geneX_B with 98% protein sequence similarity. Although variants of the same gene, they may be named geneX_A in BD-1.2 core genome (if this variant is predominant in BD-1.2) and geneX_B in BD-2 core genome (if this variant is predominant in BD-2). Comparing these independent lineages could mistakenly suggest that geneX_A is exclusive to BD-1.2 and geneX_B to BD-2, preventing further comparison. In contrast, constructing a combined pangenome, as done in our original pipeline, would correctly cluster and align these variants under a single gene name, geneX, allowing us to identify mutations (i.e. variants) specific to each lineage.

- (ii) Core vs. accessory gene classification: In separate pangenomes, some genes may be core in one lineage (because they are present in all sequences) and accessory in another (because they are present in some sequences). This would require manual correction to ensure fair comparison, increasing the risk of errors due to annotation ambiguities. On the contrary, with a combined pangenome, these genes are automatically considered accessory and correctly processed for presence/absence comparison. For example, if gene X is present in all sequences of lineage A and in some sequences of lineage B, it would be core in A and accessory in B when processed separately. Combining pangenomes ensures gene X is treated consistently as an accessory gene. If instead two pangenomes are created there is the risk that in one lineage a variant calling is done, as the gene would be erroneously considered a core gene. To illustrate this

concept, consider two lineages A and B with 100 sequences in each. If the same gene X is present in 100 sequences of linages A and in 10 sequences of lineage B, it would be considered a core gene in linage A and an accessory gene of lineage B if the pangenomes are done independently. This would require manual correction to compare across lineages as presence/absence. If instead the pangenomes are combined gene X is in 110 sequences of 200 and so automatically considered an accessory gene and processed using a presence/absence comparison across lineages.

- (iii) Core gene alignment and variant calling: Part of our analysis involves comparing mutations in genes present in the core of both BD-1.2 and BD-2. This is possible because core genes are aligned into a multi-sequence alignment file, enabling single nucleotide variant calling. With separate pangenomes, we lack a single alignment across both lineages, complicating the assessment of variants in conserved genes. Two potential workarounds include: a) Manually mapping variants by matching gene names and positions, however this is error-prone and complicated by gene naming issues (see point (i)); and b) Alternatively, aligning all sequences to a single reference genome and calling variants, then comparing across lineages. However, this method has limitations as the chosen reference genome may not represent either lineage accurately. For *V. cholerae* O1 El Tor the established reference is *V. cholerae* O1 El Tor N16961 originating from Bangladesh in 1975, which is neither from BD-2 or BD-1.2 linages. Another *V. cholerae* O1 El Tor reference strain sometimes used is C6706, but again this sequence originated from another lineage, being isolated in Peru in 1991. Due to the lineage specific difference in our isolates compared to the reference genomes from other lineages, using a reference to call the variants between BD-2 to BD-1.2 would be disadvantageous compared to the combined pangenome method employed by us. The combined pangenome approach allows for direct comparison of mutations in the two lineages, providing a more accurate analysis.

- (iv) Intergenic region comparison: Our original pipeline allowed us to cluster, align, and compare intergenic regions using Piggy, which utilized the combined pangenome output. With separate pangenomes, mapping intergenic regions across lineages would be time-consuming and error-prone, as these regions may vary in length and upstream and downstream genes.

Despite these challenges, both our combined pangenome pipeline and the lineage-specific pipeline generally align, as described below.

Construction of separate pangenomes for BD-1.2 and BD-2 isolates in our cohort.

Using Roary (Page *et al.* 2015), as in the combined pangenome analysis, we generated the pangenome for the 45 BD-2 isolates and separately for the 84 BD-1.2 isolates in our cohort (separate pangenomes**)**. Supplementary Data 6 provides the number of core, accessory, and unannotated genes in each pangenome, showing comparable numbers of core and accessory genes in both.

Difference and similarities of separate lineage-specific pangenomes compared against a single pangenome for both BD-1.2 and BD-2, using our cohort

As shown in Supplementary Data 7, the comparison of the core and accessory genes between the lineages demonstrates a significant overlap. Specifically, the same 2152 genes are present in both the BD-1.2 and the BD-2 core genomes, with 10 genes appearing as accessory in both lineages' accessory genomes. These findings match the results from our original combined pangenome covering both lineages, as the same 2152 core genes and 10 accessory genes were consistently annotated.

A small subset of genes (Supplementary Data 7) appears as core of one lineage-specific pangenome and the accessory in the other (16 accessory genes in BD-1.2 are core genes in BD-2, and 17 accessory genes in BD-2 are core genes in BD-1.2). This discrepancy is directly linked to the challenges inherent in conducting separate lineage-specific pangenomes, as discussed previously (point ii). Nevertheless, these 33 genes are all categorized as accessory in our single combined pangenome and were used in original pipeline's statistical comparison of their presence/absence.

A total of 155 genes (44 in BD-1.2 and 111 in BD-2) were found exclusively in one lineage in the separate pangenomes (Supplementary Data 7), compared to 137 (34 in BD-1.2 and 103 in BD-2) genes exclusive to a single lineage in the single pangenome pipeline. This discrepancy in gene count (i.e. the additional genes found exclusively in one lineage using separate pangenomes) arises from annotation ambiguities (see point (i) above) where variants of the same gene are named differently, thus considered distinct in lineage-specific pangenomes (see Supplementary Data 8). Hence, once the annotation ambiguities are resolved from the lineage specific pangenomes, the accessory gene set found to be exclusively present in only a single lineage in the separate pangenome analysis was identical to that found in the combined single pipeline and used as input into the statistical analysis.

In summary, in this specific study (i.e. comparing the core and accessory genome sequences across two closely related lineages) using both a combined pangenome and the lineage-specific pangenome approach yielded identical results, with the same genes considered as core and accessory, confirming that a combined pangenome approach is not bring unduly biased by population structure.

In addition, we compared the BD-1.2 and BD-2 isolates in our cohort with publicly available BD-1.2 and BD-2 sequencing data available to date, by either constructing separate pangenomes for each lineage or a combined pangenome

Construction of an extended pangenome including both our cohort and publicly available data.

To ensure that our cohort of 129 isolates was representative of the BD-1.2 and BD-2 lineages as a whole, we repeated the analysis integrating our cohort to the publicly available BD-1.2 and BD-2 sequences available to date (listed in Supplementary Data 2), used in the phylogenetic analysis shown in Figure S3, and also used in Monir *et al* 2023[2]. This gave a total of 256 isolates as input for an expanded pangenome (106 BD-1.2 and 150 BD-2 isolates) for an expanded pangenome.

In this extended (256 isolates) pangenome there are 3362 core genes of which 2148 are annotated, compared to 2183 annotated core genes in the original pangenome (129 isolates), Supplementary Data 9. Comparing between these two pangenomes we have an overlap of 2135 core genes, with only 13 additional core genes present in the extended pangenome. Four of these 13 genes were present as accessory genes in the original pangenome, and the other 9 (*rluA*, *prpF*, *dgcM*, *epsE_2*, *bcr*, *dinG*, *ddl_2*, *dgcT*, *mcpQ_2*) but named differently due to annotation ambiguities as discussed above. Comparing accessory genes, there are 156 annotated accessory genes in the original pangenome, compared to 316 annotated accessory genes in the extended pangenome as would be expected with a greater number of isolates, with an overlap of 150 genes. There were 166 additional accessory genes in the extended pangenome but only 3 of these (*hly_2*, *cat_1* and *luxO_1*) were found in significantly different distributions of BD-1.2 and BD-2 sequences, when subjected to the same statistical pipeline as employed for the original pangenome. The gene *hly_2* was also found to be statistically significant in the original

pipeline but was named *hlyA_1*. The genes *cat_1* and *luxO_1* were present in all 129 of the isolates in the original pipeline so was considered as a core gene and analysed for significant mutations, in the extended pangenome these genes were not present in 18% of BD-1.2 isolates and 17% of BD-2 isolates respectively. In total 14 annotated accessory genes from the extended pangenome were statistically significantly discriminating lineages of these 11 were found in the original analysis, 2 (*cat_1* and *luxO_1*) were instead analysed as core genes, as described above, and one, *ptsG_1* (a glucose transporter) did not meet the p-value threshold in the original pangenome analysis but was found to be statistically significant in the extended pangenome.

Overall, this analysis suggests that the original cohort of 129 isolates were largely representative of the BD-1.2 and BD-2 lineages and so the pangenome analysis to statistically compare across lineages is a valid representation of the broader behaviour of these lineages.

A single combined pangenome of 129 isolates in our cohort is representative of the wider separate BD-1.2 and BD-2 lineages

Finally, we considered how the original pipeline compared to separated lineage-specific pangenomes using the extended cohort. Considering the number of overlapping core and accessory genes in the expanded lineage-specific pangenomes (i.e. a pangenome of 106 BD-1.2 isolates and a separate pangenome of 150 BD-2 isolates), the same 2133 genes are present in both the BD-1.2 and the BD-2 core genomes, with 12 genes appearing as accessory in both lineages' accessory genome (Supplementary Data 10). These genes were also similarly found as core and accessory genes within our original cohort, except for a small number of annotation ambiguities as described above.

In total, 302 genes unique to one lineage and 43 genes core in one lineage but accessory in the other were analysed similarly as before. Of these genes, 133 were also found to be exclusively present in the analysis of our cohort alone and were tested for significance in Supplementary Data 4. A further 136 genes were found to be exclusively present in one lineage (either BD-1.2 or BD-2) in this expanded cohort of our isolates plus publicly available sequences, however all of these were present in less than 6 isolates and were not statistically significant. Of the remaining 76 genes were the results of annotation ambiguities associated with constructing separate lineage-specific genomes. Sixty-five of these were found to be core genes in the expanded pangenomes once annotation was manually corrected and were also considered as core genes within the analysis of our cohort. Similarly, 11 genes were found to be accessory genes in the expanded pangenomes after correction and were also found as accessory genes our cohort. Of note, the gene *cph2* (a phytochrome-like protein) in the expanded pangenome, whilst correctly considered as an accessory gene as identified as separating lineages compared to our initial cohort-only analysis, was not annotated in our smaller cohort. This gene had been previously reported to differentiate BD-1 and BD-2 lineages[3], which our cohort-only analysis had missed due to the lack of annotation.

This analysis confirms that our cohort is broadly representative of all available BD-1.2 and BD-2 genome sequences available to date, and further confirms that the pipeline using a combined genome, gives better results than analysing independent pangenomes which results in many false positives due to annotation ambiguities.

Supplementary Note 5:

In addition to using the generalised *V. cholerae* O1 GSM model (iAM-vc960) to analyse the metabolic functions of our strains, draft strain-specific models were generated for

each isolate using CarveMe[20]. Overall, the number of genes and reactions in the strain-specific models was slightly higher than the genes in iAM-vc960 and varied between 973 – 988 genes and 1989 – 2163 reactions, with a mean of 984 genes and 2130 reactions. The number of metabolites in the strain-specific models was lower than that of the iAM-Vc960 model with a range of 1321 – 1433 and mean 1411. The number of genes, reactions and metabolites statistically differed between BD-1.2 and BD-2 lineages (p-values < 0.0001, Mann Whitney U test).

For the lineage-based analysis, we used as input the core genome genes mapped to the non-synonymous SNPs found to be exclusively present in one lineage (i.e. present in BD-1.2 and absent in BD-2 or conversely, present in BD-2 an absent in BD-1.2). We found 14 metabolic genes which were present in the strain-specific GSM models (*clcA*, *mak*, *suhB*, *murI*, *glmM*, *appC*, *argG*, *ftsI*, *licH*, *phhA*, *dltA_1*, *putA*, *ycbB*, *hudF_2*). Of these 10 were in the generalised model (*clcA*, *mak*, *suhB*, *murI*, *glmM*, *appC*, *argG*, *ftsI*, *licH*, *phhA*) and four were not (*dltA_1*, *putA*, *ycbB*, *hudF_2*). Three genes present in the generalised model (*dsbD*, *cysG_1* and *cob*) were not present in the strain-specific models.

Comparing the gene essentiality results, *murI* was found to be essential in minimal media only in the generalised model. In the strain-specific models, it was essential in both rich and minimal media in a small proportion of models (n=16, 12%) and non-essential in both rich and minimal media in all other models. Experimental data from literature[21] shows essentiality of *murI* in the reference strain N16961 indicating some metabolic behavioural differences in our strains regarding this gene. Similarly, *glmM* was essential only in minimal media in the generalised model but essential in rich and minimal media in all the strain-specific models, consistent with its behaviour in the N16961[21]. Finally, *clcA*, which was essential in the generalised model was non-essential in all the strain-specific models, consistent with experimental results for the reference strain, where *clcA* is non-essential[21].

In the flux variability analysis, which assessed whether gene knockouts significantly alter the reaction fluxes through the GSM models, a few changes were found in the strain-specific models compared to the generalised model. Knockouts of *suhB*, *phhA* and *licH* resulted in significant flux changes in the strain-specific models but not in the generalised models. Interestingly the gene *clcA,* which generated significant flux changes in the generalised model, also caused significant flux changes in most of the BD-2 strain-specific models (91%) but only in the small proportion of the BD-1.2 models (5%).

Regarding metabolite yield changes assessed by FBA, *murI* and *mak* knockouts affected metabolite yield in the generalized model but not in strain-specific models. Conversely, *suhB* and *clcA* knockouts impacted metabolite yield in the strain-specific models but not in the generalised model.

Regarding the clinical symptom-based analysis, 11 over the genes identified by machine learning were identified as metabolic genes in the strain-specific models (*add*, *dapf, dcuH*, *gshB*, *hpt*, *pckA*, *pepN*, *cysG_2*, *padC*, *tufB*, *tufB_2*). Among these, seven were also present in the generalised model (*add*, *dapf, dcuH*, *gshB*, *hpt*, *pckA*, *pepN)* while four were *(cysG_2*, *padC*, *tufB*, *tufB_2*) unique to the strain-specific models. Two genes that were present in the generalised model (*cdgL* and *fabH1*) are absent in strain-specific models.

Comparing the gene essentiality analysis between strain-specific models and the generalised model, *dapF* was found essential in rich media for 93% of strain-specific models, whereas it was non-essential in the generalised model. This finding aligns with experimental data[21], showing this gene as essential for the reference N16961, and indicating consistency between strain-specific model results and experimental observations. In flux variability analysis, all genes showing significant flux changes in the generalized model exhibited similar changes in strain-specific models. For the

metabolites' yields analysis changes, while *dapF* knockouts showed significant metabolite yield changes in both generalized and strain-specific models, *gshB* knockouts did not show any metabolite yield changes in strain-specific models.

Supplementary Note 6:
Intergenic SNPs, exhibiting significantly different allelic distributions between BD-1.2 and BD-2, were mapped back to transcription factor binding sites of 11 TFs (*ToxT*, *Fur*, *AmpR*, *OmpR*, *LuxR*, *LexA*, *ArgR*, *PhoP*, *CRP*, *ArcA*). In particular, a binding site for Fur, the ferric uptake repressor, contained 9 intergenic SNPs (Fig. S6, S7, S10, S11 and S14). As long as there is sufficient iron present, Fur represses genes related to iron uptake and virulence in many gram-negative bacteria including *V. cholerae*[22, 23]. Two SNPs mapped to the AmpR- β-lactamase gene regulator (Fig. S8 and S10). AmpR is a LysR-type transcriptional regulator which is involved in virulence gene regulation in *V. cholerae*[24]. Another regulatory factor, *V. cholerae* OmpR, is an AphB repressor, and regulates the expression of the ToxR virulence regulon[25] (Fig. S9). Seventeen SNPs mapped in four transcription binding sites recognized by the transcription factor LuxR, a quorum sensing regulator[22,23], (Fig. S10, S14-S16). Analogously, one SNP was found in the transcription factor binding site of another quorum sensing regulator HapR, a TetR family transcription factor and homologous to LuxR[26], (Fig. S14). Three SNPs mapped into two regions recognized by the regulator of polymyxin B resistance, PhoP, (Fig. S10 and S14). Homologs of PhoP have been suggested to play a role in influencing antimicrobial resistance traits[27]. Two SNPs were found in the transcription binding motives of LexA, a master regulator of gene expression for the integration efficiency of CTXϕ in *V. cholerae*[28], (Fig. S12 and S14). Two SNPs mapped in the transcriptional binding sites of the regulator ArgR involved in arginine metabolism[29] (Fig. S13). Twenty-three SNPs mapped in the binding sites of CRP, (Fig. S14 and S16). CRP regulates *V. cholerae* virulence factors, including cholera toxin and the toxin-coregulated pilus, and some virulence-associated traits, such as motility, biofilm formation, and quorum sensing[30]. Finally, one SNP mapped in the TF binding motif of ArcA, a regulator of virulence gene expression and biofilm formation in *V. cholerae*[31] (Fig. S15).

References

1. Meng EC*, et al.* UCSF ChimeraX: Tools for structure building and analysis. *Protein Sci* **32**, e4792 (2023).

2. Monir MM*, et al.* Genomic attributes of Vibrio cholerae O1 responsible for 2022 massive cholera outbreak in Bangladesh. *Nat Commun* **14**, 1154 (2023).

3. Monir MM*, et al.* Genomic Characteristics of Recently Recognized Vibrio cholerae El Tor Lineages Associated with Cholera in Bangladesh, 1991 to 2017. *Microbiology Spectrum* **10**, e00391-00322 (2022).

4. Ramamurthy T*, et al.* Virulence regulation and innate host response in the pathogenicity of Vibrio cholerae. *Frontiers in Cellular and Infection Microbiology* **10**, 572096 (2020).

5. Sarkar A*, et al.* Altered Integrative and Conjugative Elements (ICEs) in Recent Vibrio cholerae O1 Isolated From Cholera Cases, Kolkata, India. *Front Microbiol* **10**, 2072 (2019).

6. Hounmanou YMG, *et al.* Genomic insights into Vibrio cholerae O1 responsible for cholera epidemics in Tanzania between 1993 and 2017. *PLoS Negl Trop Dis* **13**, e0007934 (2019).

7. Rogers A, Townsley L, Gallego-Hernandez AL, Beyhan S, Kwuan L, Yildiz FH. The LonA Protease Regulates Biofilm Formation, Motility, Virulence, and the Type VI Secretion System in Vibrio cholerae. *J Bacteriol* **198**, 973-985 (2016).

8. Seper A*, et al.* Extracellular nucleases and extracellular DNA play important roles in Vibrio cholerae biofilm formation. *Mol Microbiol* **82**, 1015-1037 (2011).

9. Figaj D, Ambroziak P, Rzepka I, Skórko-Glonek J. SurA-like and Skp-like Proteins as Important Virulence Determinants of the Gram Negative Bacterial Pathogens. *Int J Mol Sci* **24**, (2022).

10. Walton TA, Sousa MC. Crystal structure of Skp, a prefoldin-like chaperone that protects soluble and membrane proteins from aggregation. *Mol Cell* **15**, 367-374 (2004).

11. Hubbard TP*, et al.* Genetic analysis of Vibrio parahaemolyticus intestinal colonization. *Proc Natl Acad Sci U S A* **113**, 6283-6288 (2016).

12. Stubenrauch CJ, Lithgow T. The TAM: A Translocation and Assembly Module of the β-Barrel Assembly Machinery in Bacterial Outer Membranes. *EcoSal Plus* **8**, (2019).

13. Cakar F, Zingl FG, Moisi M, Reidl J, Schild S. In vivo repressed genes of <i>Vibrio cholerae</i> reveal inverse requirements of an H⁺/Cl[−] transporter along the gastrointestinal passage. *Proceedings of the National Academy of Sciences* **115**, E2376-E2385 (2018).

14. Cakar F, Zingl FG, Schild S. Silence is golden: gene silencing of V. cholerae during intestinal colonization delivers new aspects to the acid tolerance response. *Gut Microbes* **10**, 228-234 (2019).

15. Rothenbacher FP, Zhu J. Efficient responses to host and bacterial signals during Vibrio cholerae colonization. *Gut Microbes* **5**, 120-128 (2014).

16. Anthouard R, DiRita VJ. Small-molecule inhibitors of toxT expression in Vibrio cholerae. *mBio* **4**, (2013).

17. Behari J, Stagon L, Calderwood SB. pepA, a gene mediating pH regulation of virulence genes in Vibrio cholerae. *J Bacteriol* **183**, 178-188 (2001).

18. Anzaldi LL, Skaar EP. Overcoming the heme paradox: heme toxicity and tolerance in bacterial pathogens. *Infect Immun* **78**, 4977-4989 (2010).

19. Henderson DP, Payne SM. Vibrio cholerae iron transport systems: roles of heme and siderophore iron transport in virulence and identification of a gene associated with multiple iron transport systems. *Infect Immun* **62**, 5120-5125 (1994).

20. Machado D, Andrejev S, Tramontano M, Patil KR. Fast automated reconstruction of genome-scale metabolic models for microbial species and communities. *Nucleic Acids Res* **46**, 7542-7553 (2018).

21. Karp PD*, et al.* The BioCyc collection of microbial genomes and metabolic pathways. *Brief Bioinform* **20**, 1085-1093 (2019).

22. Woodcock SC, Raux E, Levillayer F, Thermes C, Rambach A, Warren MJ. Effect of mutations in the transmethylase and dehydrogenase/chelatase domains of sirohaem synthase (CysG) on sirohaem and cobalamin biosynthesis. *Biochem J* **330 (Pt 1)**, 121-129 (1998).

23. Watnick PI, Butterton JR, Calderwood SB. The interaction of the Vibrio cholerae transcription factors, Fur and IrgB, with the overlapping promoters of two virulence genes, irgA and irgB. *Gene* **209**, 65-70 (1998).

24. Vadlamani G*, et al.* The β-lactamase gene regulator AmpR is a tetramer that recognizes and binds the D-Ala-D-Ala motif of its repressor UDP-N-acetylmuramic acid (MurNAc)-pentapeptide. *Journal of biological chemistry* **290**, 2630-2643 (2015).

25. Kunkle DE, Bina TF, Bina XR, Bina JE. Vibrio cholerae OmpR Represses the ToxR Regulon in Response to Membrane Intercalating Agents That Are Prevalent in the Human Gastrointestinal Tract. *Infect Immun* **88**, (2020).

26. Ball AS, Chaparian RR, van Kessel JC. Quorum Sensing Gene Regulation by LuxR/HapR Master Regulators in Vibrios. *J Bacteriol* **199**, (2017).

27. Bilecen K, Fong JC, Cheng A, Jones CJ, Zamorano-Sánchez D, Yildiz FH. Polymyxin B resistance and biofilm formation in Vibrio cholerae are controlled by the response regulator CarR. *Infect Immun* **83**, 1199-1209 (2015).

28. Pant A*, et al.* Effect of LexA on Chromosomal Integration of CTXϕ in Vibrio cholerae. *J Bacteriol* **198**, 268-275 (2016).

29. Ramelot TA, Ni S, Goldsmith-Fischman S, Cort JR, Honig B, Kennedy MA. Solution structure of Vibrio cholerae protein VC0424: a variation of the ferredoxin-like fold. *Protein Sci* **12**, 1556-1561 (2003).

30. Gibson JA, Gebhardt MJ, Santos R, Dove SL, Watnick PI. Sequestration of a dual function DNA-binding protein by Vibrio cholerae CRP. *Proc Natl Acad Sci U S A* **119**, e2210115119 (2022).

31. Li Y, Yan J, Guo X, Wang X, Liu F, Cao B. The global regulators ArcA and CytR collaboratively modulate Vibrio cholerae motility. *BMC Microbiol* **22**, 22 (2022).
