# Supplementary material for: Core and accessory genomic traits of Vibrio cholerae O1 drive lineage transmission and disease severity
Source: Nat Commun. 2024 Sep 23;15:8231. doi: 10.1038/s41467-024-52238-0 (PMC11420230; doi:10.1038/s41467-024-52238-0)
Supplement: Supplementary file 3 — Description of Additional Supplementary Files [file 41467_2024_52238_MOESM3_ESM.pdf]

Description of Additional Supplementary Files

Supplementary Data 1. Characteristics of the *V. cholerae* isolates, including the sample information (organisation that collected the strain, location of the sample collection, division of the sample collection, collection date), typing data (lineage, serotype, MLST), experimental AMR profiles, presence of known AMR resistance genes (checked against the CARD database, 05-06-2022), and presence of known virulence genes (checked against VFDB database 05-06-2022). The annotation of resistance determinants may change if using a different database so alternative names for AMR genes are given in brackets according to comparison against the prokka annotation of the sequence with 90% identity and 90% coverage).

Supplementary Data 2. Accessions of the 1005 public database *V. cholerae* O1 DNA sequences used together with the 129 DNA sequences from this study. In addition to the accession, the collection country, collection year and lineage are given where available, adapted from Monir et al.[2].

Supplementary Data 3. Presence/absence of non-recombinant SNPs of 1005 public isolates together with the 129 DNA sequences from this study, with variants called against reference genome (*V. cholerae* N16961-NC_002505.1; NC_002506.1) and recombinant areas masked (see Methods). The table shows the chromosome of the reference genome, the position of the SNP, the reference nucleotide, the alternative nucleotide(s), the phred-scaled quality score and the presence/absence of the SNP in each sequence.

Supplementary Data 4. P-values from a two-sided Fisher exact test with Bonferroni correction comparing the presence/absence of features between BD-1.2 and BD-2 linages. Feature types are accessory genes, core genome coding and intergenic region SNPs. Aside from the p-value, the feature information and the percentage presence in each lineage are also provided. Bonferroni correction was applied to each feature type individually, with red-marked p-values indicating significant associations post-correction.

Supplementary Data 5. The 12 annotated accessory genes found to significantly differ between BD-1.2 and BD-2 lineages in our cohort (129 *V. cholerae* O1 isolates) and in 218 *V. cholerae* O1 reference isolates collected in Kolkata, India, and Dhaka, Bangladesh, between the years 2004 and 2022 (ENA public database http://www.ebi.ac.uk/ena). The 12 annotated genes, five – *(lon_3, endA, adh, hdfR_4* and *bcr_2)* – were predominant (over 96% presence) in BD-1.2 and absent in BD-2, and seven *(aer_3, hlyA_2, mcrC, mepM_3, mrr, tetA* and *tetR*) were present (over 97% presence) in BD-2 and absent in BD-1.2. The presence/absence patterns of the 12 genes in the BD-1.2 and BD-2 lineages in the reference isolates, align with our cohort findings.

Supplementary Data 6. Summary of core, accessory, and unannotated gene counts in the pangenomes for the 84 BD-1.2 isolates and the 45 BD-2 isolates in our cohort.

Supplementary Data 7. Summary of overlapping and non-overlapping genes in the core and accessory sets of the pangenomes for the 84 BD-1.2 isolates and the 45 BD-2 isolates in our cohort.

Supplementary Data 8. Genes found in the separate lineage specific pangenomes of either BD-1.2, BD-2 showing the annotation problems and how these genes map to the initial cohort-only analysis.

Supplementary Data 9. Summary of core, accessory, and unannotated gene counts in the pangenomes for 106 BD-1.2 isolates (84 from our cohort and 22 publicly available genomes, see Dataset 2) and 150 BD-2 isolates (45 from our cohort and 105 publicly available genomes, see Dataset 2) and the combined BD-1.2 and BD-2 pangenome (256 isolates).

Supplementary Data 10. Summary of overlapping and non-overlapping genes in the core and accessory sets of the pangenomes for 106 BD-1.2 isolates (84 from our cohort and 22 publicly available genomes, see Dataset 2) and 150 BD-2 isolates (45 from our cohort and 105 publicly available genomes, see Dataset 2).

Supplementary Data 11. Summary of GSMM analysis, using the model iAM-Vc960, of the core genes underlying the BD-1.2 and BD-2 lineages separation. For each gene, the molecular functions, systems and subsystems, essentiality on rich and minimal media, the autotropic behaviour, essentiality on alternative carbon sources and significance in FVA and FBA analysis are detailed. For the FVA analysis a gene was significant, if when knocked out, the flux span across at least one reaction changed by > 10% compared to wildtype. For the FBA analysis a gene was significant, if when knocked out, at least one metabolite yield reduced to 0, given that the yield was greater than 0 in the wildtype.

Supplementary Data 12. Summary of GSMM analysis, using the strain-specific models, of the core genes underlying the BD-1.2 and BD-2 lineages separation. For each gene, the molecular functions, pathways and biological processes, essentiality on rich and minimal media, the autotropic behaviour, essentiality on alternative carbon sources and significance in FVA and FBA analysis are detailed. For the FVA analysis a gene was significant, if when knocked out, the flux span across at least one reaction changed by > 10% compared to wildtype. For the FBA analysis a gene was significant, if when knocked out, at least one metabolite yield reduced to 0, given that the yield was greater than 0 in the wildtype.

Supplementary Data 13. P-values from a two-sided chi-square test of independence to measure the dependency between the clinical symptoms' phenotypes (abdominal pain, vomit, number of stools 11-15 times vs. 21+ times, number of stools 11-15 times vs. 16-20 times, dehydration moderate vs severe and diarrhoea duration <1day vs 1-3 days) and the confound effects (sex of patient, age of patient, location of patient, year of collection and serology of *V. cholerae*). Red-marked p-values indicate significant associations post-correction.

Supplementary Data 14. Clinical symptom and demographic data of hospitalised patients from whom the isolates and metadata were collected. Isolate lineage, collection date, patient age, and sex are given as well as the presence or severity of 5 clinical symptoms: duration of diarrhoea, number of stools recorded of 24-hour period, presence/absence of abdominal pain, presence absence of vomiting, dehydration (clinical assessment).

Supplementary Data 15. Machine learning performance indicators for each of the seven classification methods used to investigate if correlations exist between the genomic determinants of the BD-1.2 isolates and clinical manifestations among hospitalised patients from whom the isolates were collected from. The performance metrics were accuracy (TP+TN/(P+N)), sensitivity (true positive rate: TP/P), specificity (true negative rate: TN/N), AUC and precision. The scores for each performance metric were computed from 30 simulations using nested cross-validation. The mean ± standard deviation of these 30 iterations was then used as the result statistics for the performance.

Supplementary Data 16. The significant features selected by the machine learning pipeline as predictive of clinical symptoms. For each feature, the type, predictive models in which it was selected, the corresponding gene name (or intergenic region, showing the flanking upstream and downstream genes and the distances between the downstream and the upstream genes) and protein name. Features were highlighted in yellow if underlying the BD-1.2 and BD-2 lineages separation.

Supplementary Data 17. Summary of GSMM analysis, using the model AM-Vc960, of the core genes found to be predictive of clinical symptoms. For each gene, the molecular functions, systems and subsystems, essentiality on rich and minimal media, the autotropic behaviour, essentiality on alternative carbon sources and significance in FVA and FBA analysis are detailed. For the FVA analysis a gene was significant, if when knocked out, the flux span across at least one reaction changed by > 10% compared to wildtype. For the FBA analysis a gene was significant, if when knocked out, at least one metabolite yield reduced to 0, given that the yield was greater than 0 in the wildtype.

Supplementary Data 18. Summary of GSMM analysis, using the strain-specific models, of the core genes found to be predictive of clinical symptoms. For each gene, the molecular functions, systems and subsystems, essentiality on rich and minimal media, the autotropic behaviour, essentiality on alternative carbon sources and significance in FVA and FBA analysis are detailed. For the FVA analysis a gene was significant, if when knocked out, the flux span across at least one reaction changed by > 10% compared to wildtype. For the FBA analysis a gene was significant, if when knocked out, at least one metabolite yield reduced to 0, given that the yield was greater than 0 in the wildtype.

Supplementary Data 19. Functional annotation of each ML selected gene associated to clinical symptoms. Annotations were generated by String DB[2] and categorised as biological processes (BP), metabolic functions (MF), cellular components (CC) and KEGG terms.

Supplementary Data 20. Gene set enrichment analysis of the 36 clinical symptoms-associated genes and the 109 other interacting proteins (found by the PPI). Shown are significantly enriched biological processes, metabolic functions, cellular components and KEGG terms. An FDR adjusted p-value of 0.05 was used to infer significant sets.

Supplementary Data 21. Primers used to biotype presumptive *V. cholerae* isolates.
