# Supplementary material for: Core and accessory genomic traits of Vibrio cholerae O1 drive lineage transmission and disease severity
Source: Nat Commun. 2024 Sep 23;15:8231. doi: 10.1038/s41467-024-52238-0 (PMC11420230; doi:10.1038/s41467-024-52238-0)
Supplement: Supplementary file 25 — Reporting Summary [file 41467_2024_52238_MOESM25_ESM.pdf]

nature portfolio

Corresponding author(s): Tania Dottorini

Last updated by author(s): Aug 13, 2024

Reporting Summary

Nature Portfolio wishes to improve the reproducibility of the work that we publish. This form provides structure for consistency and transparency in reporting. For further information on Nature Portfolio policies, see our Editorial Policies and the Editorial Policy Checklist.

Statistics

For all statistical analyses, confirm that the following items are present in the figure legend, table legend, main text, or Methods section.

n/a	Confirmed	
☐	☒	The exact sample size (*n*) for each experimental group/condition, given as a discrete number and unit of measurement
☐	☒	A statement on whether measurements were taken from distinct samples or whether the same sample was measured repeatedly
☐	☒	The statistical test(s) used AND whether they are one- or two-sided<br>*Only common tests should be described solely by name; describe more complex techniques in the Methods section.*
☒	☐	A description of all covariates tested
☐	☒	A description of any assumptions or corrections, such as tests of normality and adjustment for multiple comparisons
☐	☒	A full description of the statistical parameters including central tendency (e.g. means) or other basic estimates (e.g. regression coefficient) AND variation (e.g. standard deviation) or associated estimates of uncertainty (e.g. confidence intervals)
☐	☒	For null hypothesis testing, the test statistic (e.g. *F*, *t*, *r*) with confidence intervals, effect sizes, degrees of freedom and *P* value noted<br>*Give P values as exact values whenever suitable.*
☒	☐	For Bayesian analysis, information on the choice of priors and Markov chain Monte Carlo settings
☒	☐	For hierarchical and complex designs, identification of the appropriate level for tests and full reporting of outcomes
☒	☐	Estimates of effect sizes (e.g. Cohen's *d*, Pearson's *r*), indicating how they were calculated

Our web collection on statistics for biologists contains articles on many of the points above.

Software and code

Policy information about availability of computer code

Data collection

N/A

Data analysis

The code developed and used by us to analyse the data in this study has been made open source and is available on Github: https://github.com/tan0101/VibrioCARE

This link has also been added in the "Code availability" section of the manuscript

For the bioinformatics data analysis, the following open-source software were used:
Illumina BaseSpace sequencing hub
SPAdes v3.12
ContEst16S
Prokka v1.14.6
Roary v3.13
SNP sites 2.5.1
Piggy v1.5
IQ Tree v2.2.0.3
iTol v6
Snippy v4.6.0
Gubbins v.2.3.4
Clustal Omega v1.2.4

Jalview 2.11.3.2
BPROM/softberry
cobra toolkit v0.26.2
CarveMe v1.6.1
cplex v20.1
cameo toolbox v0.13.6
Cytoscape v3.10.1
UCSF Chimera
UCSF ChimeraX
dDUET
DynaMut
SIFT

To perform the machine learning (ML) analysis the following software were used:
python v3.9.15
scikit-learn v1.2.1
scipy v1.9.3
networkx v2.8.4
Matplotlib v3.6.2

The bioinformatics and Machine learning software listed above were properly mentioned and cited in the manuscript.

For manuscripts utilizing custom algorithms or software that are central to the research but not yet described in published literature, software must be made available to editors and reviewers. We strongly encourage code deposition in a community repository (e.g. GitHub). See the Nature Portfolio guidelines for submitting code & software for further information.

Data

Policy information about availability of data

All manuscripts must include a data availability statement. This statement should provide the following information, where applicable:

- Accession codes, unique identifiers, or web links for publicly available datasets
- A description of any restrictions on data availability
- For clinical datasets or third party data, please ensure that the statement adheres to our policy

Short-read sequence data for all 129 isolates used in this study are deposited in the NCBI SRA and can be found associated with BioProject number PRJNA1021874 [https://www.ncbi.nlm.nih.gov/bioproject/?term=PRJNA1021874]. All previously published public V. cholerae sequences used in this study are held in European Nucleotide Archive-ENA or NCBI repositories under accession numbers supplied in Supplementary Data 2. Reference sequences are available from NCBI under accessions: NC_002505.1 [https://www.ncbi.nlm.nih.gov/nuccore/NC_002505.1], NC_002506.1 [https://www.ncbi.nlm.nih.gov/nuccore/NC_002506.1] and European Nucleotide Archive-ENA under accession: ERR025382 [https://www.ebi.ac.uk/ena/browser/view/ERR025382]. Clinical data used in this study is given in Supplementary Data 14.

All these links are reported in the manuscript under the section: "Data availability"

Research involving human participants, their data, or biological material

Policy information about studies with human participants or human data. See also policy information about sex, gender (identity/presentation), and sexual orientation and race, ethnicity and racism.

Reporting on sex and gender	The sex of the participants was reported in Dataset S13. The findings of this paper did not consider a sex separation.
Reporting on race, ethnicity, or other socially relevant groupings	The study did not collect or analyse data on race, ethnicity or other socially relevant groupings.
Population characteristics	The isolates included in the study were gathered from patients meeting the case definition of diarrhoea. The case definition of diarrhoea was standardized to ensure consistency across different regions and over the collection timeline. In addition, subsequent to sample collection, isolates were confirmed as V. cholerae using classical microbiological and molecular biology analysis (see methods) and whole genome sequencing.
Recruitment	The isolates included in the study were gathered from patients meeting the case definition of diarrhoea and consenting to be included in the surveillance study. Confirmed V. cholera cases were used in this study. Only the whole-genome sequencing of the V. cholera isolates were used in the study. Clinical data covered 5 categories (duration of diarrhoea, number of stools, abdominal pain, vomiting, and dehydration), in addition the age and sex of the patient and location of the patient was recorded.
Ethics oversight	Informed written consent was obtained from all adult patients, or guardians on behalf of children. Upon receiving consent, the physician collected the patient's sociodemographic characteristics and medical histories. For the icddr,b isolates, the study protocol was approved by the Institutional Review Board of icddr,b (PR-15127). For the IEDCR isolates, the study was performed in accordance with protocols approved by the Institutional Review Board of IEDCR (IEDCR/IRB/09 and IEDCR/IRB/26). Ethics approval was also obtained from the School of Veterinary Medicine and Science Ethics Committee University of Nottingham (2811 110724).

Note that full information on the approval of the study protocol must also be provided in the manuscript.

Field-specific reporting

Please select the one below that is the best fit for your research. If you are not sure, read the appropriate sections before making your selection.

☒ Life sciences ☐ Behavioural & social sciences ☐ Ecological, evolutionary & environmental sciences

For a reference copy of the document with all sections, see nature.com/documents/nr-reporting-summary-flat.pdf

Life sciences study design

All studies must disclose on these points even when the disclosure is negative.

Sample size	For the analysis of how to separate the lineages of V. cholerae (in relation to different genomic features) we did not perform sample size calculation as this was an observational/exploratory study.<br><br>For the ML analysis to discriminate the different clinical symptoms connected to the BD-1.2 isolates, we performed a sample size calculation as detailed in the following. For the results demonstrating the proposed approach to the clinical symptom prediction, sample size is based on achieving desired power in the predictor. For binary classifiers, power is the sensitivity (true positive rate, defined as 1-beta, where beta is the false negative rate, i.e. type II error (Banerjee, A. et al. 2009, Industrial psychiatry journal). Using 63 V. cholerae isolates from lineage BD-1.2, we achieved an average power of 83% for the 6 clinical symptom models studied. We also wanted to identify the minimum number of samples required to achieve at least 80% sensitivity (power). Because for classifiers based on ML (e.g. SVMs, decision trees, random forest, adaboost, neural networks), sample size calculation to achieve power is not directly possible using conventional analytical methods (Li. J. et al. 2020, Patterns), we applied a bespoke iterative method (wrapper backward selection - WBS, Figueroa, R et al. 2012, BMC Medical Informatics and Decision Making) as done in our previous paper (Maciel-Guerra, A. et al. 2022, The ISME Journal; and Peng et al. 2022, Plos Computational Biology). The method estimates how power decreases with smaller sample sizes. In our case, WBS estimated the need of 56 isolates on average, which is less to what we used.
Data exclusions	No data were excluded.
Replication	n/a
Randomization	Biological samples were collected randomly from patients with diarrhoea symptoms without knowing the V. cholera lineage<br>For the ML classification the samples were randomly assigned to training and testing groups using a nested cross validation procedure (30 iterations per classifier).
Blinding	Biological samples were collected randomly from patients with diarrhoea symptoms without knowing the V. cholera lineage

Reporting for specific materials, systems and methods

We require information from authors about some types of materials, experimental systems and methods used in many studies. Here, indicate whether each material, system or method listed is relevant to your study. If you are not sure if a list item applies to your research, read the appropriate section before selecting a response.

Materials & experimental systems

n/a	Involved in the study	
☒	☐	Antibodies
☒	☐	Eukaryotic cell lines
☒	☐	Palaeontology and archaeology
☒	☐	Animals and other organisms
☒	☐	Clinical data
☒	☐	Dual use research of concern
☒	☐	Plants

Methods

n/a	Involved in the study	
☒	☐	ChIP-seq
☒	☐	Flow cytometry
☒	☐	MRI-based neuroimaging

Plants

Seed stocks	n/a
Novel plant genotypes	n/a
Authentication	n/a
